# Bioinformatic characterization of the Anoctamin Superfamily of Ca^2+^-activated ion channels and lipid scramblases

**DOI:** 10.1371/journal.pone.0192851

**Published:** 2018-03-26

**Authors:** Arturo Medrano-Soto, Gabriel Moreno-Hagelsieb, Daniel McLaughlin, Zachary S. Ye, Kevin J. Hendargo, Milton H. Saier

**Affiliations:** 1 Department of Molecular Biology, University of California at San Diego, La Jolla, California, United States of America; 2 Department of Biology, Wilfrid Laurier University, Waterloo, Ontario, Canada; Eye Hospital, Charité, GERMANY

## Abstract

Our laboratory has developed bioinformatic strategies for identifying distant phylogenetic relationships and characterizing families and superfamilies of transport proteins. Results using these tools suggest that the Anoctamin Superfamily of cation and anion channels, as well as lipid scramblases, includes three functionally characterized families: the Anoctamin (ANO), Transmembrane Channel (TMC) and Ca^2+^-permeable Stress-gated Cation Channel (CSC) families; as well as four families of functionally uncharacterized proteins, which we refer to as the Anoctamin-like (ANO-L), Transmembrane Channel-like (TMC-L), and CSC-like (CSC-L1 and CSC-L2) families. We have constructed protein clusters and trees showing the relative relationships among the seven families. Topological analyses suggest that the members of these families have essentially the same topologies. Comparative examination of these homologous families provides insight into possible mechanisms of action, indicates the currently recognized organismal distributions of these proteins, and suggests drug design potential for the disease-related channel proteins.

**List of Abbreviations**

**Families**

ANO: Anoctamin (TC: 1.A.17.1)
ANO-L: Anoctamin-like (TC: 1.A.17.2)
CSC: Calcium-permeable Stress-gated Cation Channel (TC: 1.A.17.5)
CSC-L1: Calcium-permeable Stress-gated Cation Channel-like 1 (TC: 1.A.17.3)
CSC-L2: Calcium-permeable Stress-gated Cation Channel-like 2 (TC: 1.A.17.7)
TMC: Transmembrane Channel (TC: 1.A.17.4)
TMC-L: Transmembrane Channel-like (TC: 1.A.17.6)

**Programs**

AveHAS: program for determining Average Hydropathy, Amphipathicity and Similarity for a set of multiply aligned homologous sequences
GSAT: Global Sequence Alignment Search Tool
ITOL: Interactive Tree of Life, a web-based environment for the display of phylogenetic trees
MAFFT: a program for creating multiple sequence alignments
mkProteinClusters.pl: program for clustering protein sequences based on bit scores derived from BLASTP, SSEARCH36, FASTA36 or UBLAST
MrBayes: a program for building phylogenetic trees
Phylip: a suite of programs for phylogenetic analysis
SuperfamilyTree: program for constructing protein trees using BLAST bit scores rather than multiple alignments
WHAT: Web-based program for determining Hydropathy, Amphipathicity and Topology for single proteins

**Other**

aas: amino acyl residues
CDD: Conserved Domain Database
DUF: Domain of Unknown Function
SD: Standard Deviation
TCDB: Transporter Classification Database
TMS: Transmembrane Segment

## Introduction

In January of 1993, our laboratory reported bioinformatic studies that provided the first evidence suggesting an evolutionary relationship among drug resistance exporters, glucose facilitators, metabolite uptake proteins, sugar phosphate antiporters, and the well-studied lactose permease of *Escherichia coli* [1]. We named this superfamily the Major Facilitator Superfamily (MFS). In subsequent publications, we identified many more members of this superfamily [2–5]. In 2016, there were nearly 100 families in the MFS, and our most recent unpublished efforts have identified additional MFS family members. Moreover, it appears that transmembrane peptidases and glycosyltransferases may also be members of this superfamily (S. Wang, I. Javadi-Razat and M.H. Saier, unpublished results). The MFS is now the largest superfamily of transmembrane transporters currently recognized. Proposals for the pathways of its evolution have been presented [6–8], and comparison of high resolution x-ray structures support these proposals [9–12].

Since the identification of the MFS, our laboratory has identified over 60 superfamilies of transport proteins (see the Superfamily Hyperlink in the Transporter Classification Database—TCDB: tcdb.org). The largest superfamily of ion channels is the Voltage-gated Ion Channel (VIC) Superfamily (TC: 1.A.1) [13–15], and the largest superfamily of primary active transporters is the ATP-binding Cassette (ABC) Superfamily (TC: 3.A.1) [16], which actually includes at least three, and possibly as many as six, evolutionarily distinct families of integral membrane transport proteins [17–19]. Our bioinformatic strategies have become increasingly sensitive and refined over the past years. Here, we use these strategies to define, expand and organize a novel superfamily, the Anoctamin (ANO) Superfamily, which, after the analyses reported here, includes 7 families, three of known function and four of unknown function. The bioinformatically-derived characteristics of the included proteins are described.

### Anoctamins (TC: 1.A.17.1)

Anoctamins, also referred to as TMEM16 proteins, comprise a family of proteins that mediate ion transport, phospholipid scrambling, and regulation of other membrane proteins [20–24]. Ano1 and Ano2 play roles in transepithelial ion transport, smooth muscle contraction, olfaction, phototransduction, nociception, heat sensitivity and control of neuronal excitability [21, 22, 25, 26]. Mutations in these human anoctamins have been found to be associated with disease conditions including muscular dystrophies, febrile seizures and cerebellar ataxia [27–31]. Additionally, Ano5, has been implicated in muscle and bone diseases [32–34], Ano6 is important for innate immunity, and mutations in Ano6 cause Scott Syndrome (a bleeding disorder) [35, 36], while Ano10 may play a role in macrophage volume regulation [37]. Ano1 has been reported to be the major apical iodide channel in thyrocytes [30, 38, 39]. Further, overexpression of the genes encoding Ano1 and Ano3 have been linked to several forms of cancer, specifically to gastrointestinal stream tumors, breast cancers, and squamous cell carcinomas [27, 40]. Ano4 regulates aldosterone secretion in the zona glomerulosa of the human adrenal gland [41]. Several anoctamins, most notably Ano6, have been shown to be phospholipid scramblases, facilitating phosphatidyl serine translocation from the inner leaflet of the plasma membrane to the other leaflet [42–44], a process that can signal apoptosis although XKR8 is the apoptotic caspase-regulated scramblase [45]. Some TMEM16 homologues, including the *Nectria haematococcus* homologue, nhTMEM16, exhibit both ion channel and lipid scramblase activities [21, 46–48]. It has recently been shown that mutation of a couple of residues in the subunit cavity of TMEM16A convert the Cl^-^ channel into a scramblase [21, 46].

Anoctamins are present in numerous eukaryotes that have been examined for these proteins with 10 paralogs identified in vertebrates named Ano1 through Ano10 (TMEM16A-H, THEM16J and K, respectively) [49], and several have been shown to be Ca^2+^-activated Cl^-^ channels (CaCCs). It was originally proposed that Ano1 and Ano2 have an 8-transmembrane segment (TMS) topology with a re-entrant loop between the fifth and sixth TMSs [49], but this proposal is now known to be incorrect [50, 51]. X-ray structural data for one homologue from the fungus, *Nectria haematococcus*, and cryoEM data for mouse Ano1 support a 10 TMS model lacking a reentrant loop [20, 48, 52, 53]. The potential relationship of this structure to the functions of ion transport and lipid flipping has been discussed [20, 48]. The name “Anoctamin” was given to this protein family prior to its structural elucidation as a result of the originally proposed 8 TMS topology and the anion (Cl^-^, HCO_3_^-^, I^-^, NO_3_^-^, SCN^-^, F^-^, etc.) conductances expressed by Ano1 and Ano2 (anion = ano; 8 = oct) [27, 54]. In spite of the facts that members of the superfamily may have up to 10 TMSs, and some catalyze cation rather than anion transport in addition to scrambling phospholipids, the term “anoctamin” appears to be thoroughly entrenched in the scientific literature. It brings up 2.5 times as many publications in PubMed as the alternative term TMEM16, and 4.5 times as many as the term, transmembrane channel or TMC. Hence, in this paper, the term “anoctamin” will be retained.

Anoctamin regulation has been extensively studied [51, 55–57], yet the mechanisms by which an increased intracellular Ca^2+^ concentration activates chloride or cation conductance and phospholipid flippase activity are still poorly understood [58]. Early studies indicated that calmodulin, a Ca^2+^ binding protein, is required for this process, but the reported effect of calmodulin may have been indirect [59]. More recent studies have shown that the purified Ano1 protein alone is sufficient to mediate Ca^2+^-activation. Neither calmodulin, nor any other accessory protein is required for channel activation by either Ca^2+^ or voltage [46, 60–63].

A set of two conserved glutamate residues between putative TMSs 6 and 7 have been suggested to be responsible for Ano1 activation by Ca^2+^ [50, 51, 63]. On the other hand, Galietta noted that anoctamins contain a series of 5 consecutive glutamate residues that are located in the region between putative TMSs 2 and 3, and that these residues could be a site of both Ca^2+^ sensitivity and voltage-dependent activation [64]. However, Tien et al. [63], identified five other acidic residues in the second half of the protein that appeared to be critical for Ca^2+^ sensitivity. Yang et al. presented evidence that a K584Q mutation in TMEM16A/Ano1 (residue 559 in TMEM16F), alters the anion/cation selectivity [43], but this result could not be reproduced in a subsequent study [65]. Although the reasons for this discrepancy are unclear, the evidence available suggests that residues facing the channel pore control both ion selectivity and gating of the channel [66].

Wild type Anoctamin channels, Ano1 and Ano2, in the presence of a sub-optimal Ca^2+^ concentration will activate upon imposition of a positive membrane potential, and deactivation occurs when the membrane potential returns to its resting state [54, 67, 68]. When the Ca^2+^ concentration is at optimal levels, the channel becomes active at negative membrane potentials [69]. Splice variants of anoctamins have different levels of voltage and Ca^2+^ concentration dependencies as well as ion selectivities [70, 71].

As noted above, other anoctamins have been examined for their transport functions and physiological impacts. Most have been reported to be ion channels and/or phospholipid scramblases, and some are believed to regulate other channels [21, 35]. Ano6 may act indirectly in bone mineralization by activating the calcium transporter, NCX1 [72]. Ano10 may function in volume regulation in macrophages [37], while Ano5 may be responsible for Limb-girdle muscular dystrophies [32, 73, 74]. High-resolution structures of the fungal nhTMEM16 homologue are available, and the residues that bind Ca^2+^ as well as the subunit cavity used for scrambling phospholipids have been identified, but major questions regarding the mechanisms of ion and phospholipid translocation still remain [20, 48, 75].

### Transmembrane Channel-like (TMC) proteins (TC: 1.A.17.4)

Through sequence similarity, the transmembrane channel (TMC) proteins have been suggested to be homologous to anoctamins [76–78]. TMC proteins had also been predicted to have an 8 TMS topology, as suggested for anoctamins, but as noted above, the x-ray data for the fungal member of the Anoctamin superfamily, nhTMEM16, does not support this model [20, 48]. Several conserved amino acyl residues (aas) have been identified in putative TMSs 4–7 that correspond in position and nature to residues in the hydrophobic regions of the anoctamins [78]. TMC homologues have been studied primarily in animals, although homologues have been found in other eukaryotic phyla (see TCDB and Table 1). Their organismal distribution differs from the species diversity recognized for the anoctamins.

**Table 1:** Average protein sizes, numbers of predicted TMSs (based on average hydropathy plots) and source phyla for each of the seven major families in the Anoctamin Superfamily.

Family	TC Id	Average protein size (aas)	Average number of hydrophobic peaks	Organismal phyla
**ANO**	**1.A.17.1**	897 ± 155	9	Metazoa, Albunigaceae, Saprolegniaceae, Phaeophyceae, Salpingoecidae, Ichthyosporea,
**ANO-L**	**1.A.17.2**	994 ± 134	8	Metazoa
**TMC**	**1.A.17.4**	835 ± 143	9	Metazoa, Salpingoecidae, Viridiplantae, Ichthyosporea
**TMC-L**	**1.A.17.6**	841 ± 101	9	Intramacronucleata, Peronosporales, Phaeophyceae, Cryptophyta
**CSC**	**1.A.17.5**	774 ± 36	10	Metazoa, Viridiplantae, Fungi
**CSC-L1**	**1.A.17.3**	903 ± 106	9	Metazoa, Viridiplantae, Fungi, Saprolegniaceae, Phaeophyceae, Pelagophycea, Oligohymenophorea, Bacillariophyta, Spirotrichea, Eustigmatophyceae
**CSC-L2**	**1.A.17.7**	703 ± 124	9	Hexamitidae

There are 8 TMC paralogs in animals named TMC1 through TMC8. Mutations in TMC1, the best studied TMC, cause deafness in both mice and humans and reduce Ca^2+^ permeability [79, 80]. It has been shown that mice lacking a functional TMC1 fail to develop working cochlear neurosensory hair cells [81]. TMC1 and TMC2 expressed in these cells are crucial for mechanotransduction, where Ca^2+^ enters the cell in response to sound vibrations [82]. TMC gene therapy has been shown to restore auditory function in deaf mice [83]. Some TMCs may allow transmembrane flow of Ca^2+^, Zn^2+^, and possibly other cations [84].

Additional experiments have elucidated possible functions for TMC1 and its homologues. TMC1 acts as a sensor for salt chemosensation in *Caenorhabditis elegans* and is required for behavioral avoidance in response to increased NaCl concentrations [85]. It plays a role in *C*. *elegans* development and sexual behavior. Expression of *C*. *elegans* TMC1 in mammalian cell cultures resulted in Na^+^-activated cation conductance. These data suggest a possible function for TMC1 as an ionotropic receptor [85]. Functions of TMCs 3–8 are less well understood, although TMC 6 and 8 are implicated in the human disease, epidermodysplasia verruciformis, which involves increased susceptibility to human papilloma virus infection [86].

### Calcium-permeable Stress-gated Cation Channel (CSC) proteins (TC: 1.A.17.5)

Another family that has been associated with the Anoctamin Superfamily has been designated the RSN1_7TM Family, previously known as DUF221, where DUF stands for “Domain of Unknown Function” [87]. Several of these proteins are osmosensitive Ca^2+^-permeable cation channels [88]. Hou et al. initially characterized an RSN1_7TM homologue from *Arabidopsis thaliana*. This homolog proved to be a non-rectifying, plasma membrane, calcium permeable, stress-gated, cation channel which they designated CSC1 (TC: 1.A.17.5.10) [88]. It was a 771 amino acyl residue (aa) protein predicted to have nine TMSs plus a reentrant loop between putative TMSs 6 and 7, a prediction no longer likely to be correct (see above and below). It was activated by hyperosmotic shock and proved to be permeable to Ca^2+^, K^+^ and Na^+^. Inactivation or channel closure was Ca^2+^-dependent. Bioinformatic analyses suggested the presence of 3 N-terminal TMSs, the first of which was considered to be a cleavable signal peptide. The C-terminal region of 6 putative TMSs corresponded to the RSN1_7TM domain. *Arabidopsis* species contain at least 15 CSCs [88], and some of the genes encoding the various plant homologues are transcriptionally upregulated in response to various abiotic and biotic stresses involving mechanical perturbation [89].

Hou et al. also characterized a CSC1 protein from the yeast, *Saccharomyces cerevisiae*, one of four paralogues in this organism [88]. This channel was activated under hyperosmotic conditions. This research group also characterized a CSC1 homologue in humans, and as expected, it too proved to be activated by hyperosmolarity and Ca^2+^ [88]. The authors therefore characterized three presumed orthologues, one from a plant, one from a fungus, and one from an animal, all exhibiting similar cation channel properties regulated by essentially the same stimuli.

In this communication, we conclude that these three families (ANO, TMC, and CSC) as well as four previously unidentified families (ANO-L, TMC-L, CSC-L1, and CSC-L2) are members of the newly defined Anoctamin Superfamily. We provide the characteristics of the proteins that comprise each of these seven families (see the superfamilies link in TCDB).

## Results

As a result of the analyses reported below, within 1.A.17, the Anoctamin (ANO) family is represented by the identifier 1.A.17.1, TMC is represented by 1.A.17.4, and CSC is represented by 1.A.17.5. The four families consisting of proteins of unknown function were given the identifiers 1.A.17.2 (designated the ANO-like or ANO-L Family), 1.A.17.6 (designated the TMC-like or TMC-L Family), 1.A.17.3 (designated the CSC-like 1 or CSC-L1 Family), and 1.A.17.7 (designated the CSC-like 2 or CSC-L2 Family).

### Family expansion

This work started by considering six families (TC: 1.A.17.1 to 1.A.17.6). Each of the original six families was extended with our program findDistantFamilyHomologs (see Methods) to incorporate divergent proteins. As a result of this expansion, an additional small family was identified (CSC-L2; TC: 1.A.17.7). The CSC-L2 family consists of proteins of 600–850 aas with at least 9 putative TMSs. These proteins are found in organisms from the Hexamitidae taxonomic family, including microscopic free living and pathogenic flagellated protozoa of the *Giardia* and *Spironucleus* genera [90].

### Conserved domains

**Fig 1:**
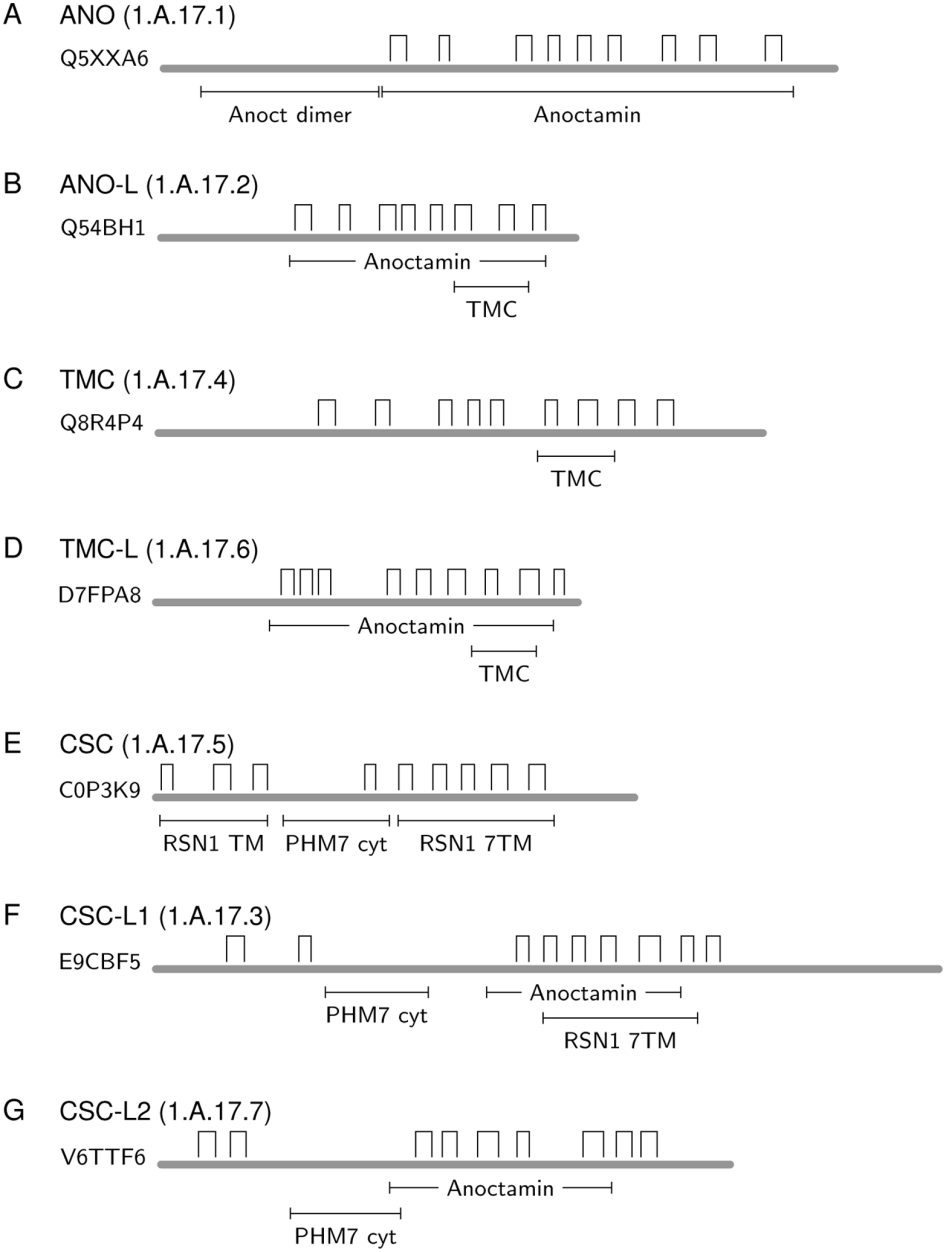
Predicted topologies and domain organizations of various members of the Anoctamin Superfamily. Open rectangular bars denote the positions of hydrophobic peaks, indicating putative TMSs. The locations of recognized Pfam domains are shown below thick gray lines representing the protein sequences.

Results of querying Pfam [91] with members of the Anoctamin Superfamily were used to study domain architectures for each family within the Anoctamin Superfamily (Fig 1). Seven families (TC: 1.A.17.1-1.A.17.7) have different combinations of recognizable Pfam domains. The main domain in each family was present in all members, while secondary domains were not always identified in all members (see Methods). The predicted TMSs and domain arrangements of the seven families (Fig 1A–1G) in the Anoctamin Superfamily showed distinct, but often overlapping, domains. Three of the dominant domain designations, “Anoctamin”, “TMC” and “RNS1-7TM” overlap and are part of the same Pfam clan Anoctamin-like (CL0416), and thus suggest homologous, albeit divergent motifs (Fig 1).

In the Anoctamin family (Fig 1A; TC: 1.A.17.1), a large Anoctamin domain was recognized that covered all putative TMSs [49]. A hydrophilic, N-terminal Anoctamin dimerization domain was also identified. The ANO-L family proteins (Fig 1B, TC: 1.A.17.2) included two overlapping Pfam domains: an Anoctamin domain encompassing all TMSs, and a C-terminal TMC domain encompassing 3 putative TMSs. This observation suggests that the short TMC domain is part of the full length Anoctamin domain (compare Fig 1A with Fig 1B).

TMC proteins (Fig 1C; TC: 1.A.17.4) only matched the TMC domain that contains three predicted TMSs near the C-terminus, while TMC-L family members (Fig 1D; TC 1.A.17.6) showed a domain architecture similar to that of ANO-L (compare with Fig 1B).

The CSC Family (Fig 1E; TC: 1.A.17.5) contains three domains, an N-terminal RSN1 TM domain (spanning putative TMSs 1–3), a central cytoplasmic PHM7 cyt domain (of unknown function), and a C-terminal RSN 7TM domain (spanning putative TMSs 5–9). The RSN1 domains are defined as Ca^2+^-dependent channel domains, clearly reflective of their associations with functionally characterized members of the Anoctamin Superfamily. The domain organization of the CSC-L1 family (Fig 1F; TC: 1.A.17.3) also matched the cytoplasmic PHM7 cyt domain. This family shows overlap between the RSN1 7TM domain and the Anoctamin domain, revealing the equivalence of these two distantly related domains. RSN1 TM has an unknown function, but experiments in yeast have shown that Sro7P-deficient mutants, defective in a protein containing this domain, exhibit increased sensitivity to NaCl concentrations because Sro7P, a large soluble protein that is unrelated to any member of the Anoctamin superfamily, is responsible for localizing sodium pumps to the cell membrane in order to remove excess Na^+^ from the cytoplasm. Overexpression of Sro7P has been shown to re-route these sodium pumps to the plasma membrane, restoring NaCl tolerance [92]. The presence of these three domains in nearly all CSC proteins suggests that the three domains function together. The functions of uncharacterized CSC proteins are likely to correspond to those of the three characterized members of the family [88].

Finally, the CSC-L2 family proteins (Fig 1G; TC: 1.A.17.7) exhibit the cytoplasmic PHM7 cyt domain and the Anoctamin domain, thus displaying a domain architecture similar to those of the CSC and CSC-L1 families. BLAST searches against TCDB show that CSC-L2 family members are more similar to proteins in the CSC and CSC-L1 families.

The Pfam domain matches thus suggest that all the families examined are members of a superfamily. The results in this section were confirmed by NCBI’s Conserved Domain Database (CDD) [93] matches obtained using rpsblast with composition-based statistics and masking low-information regions.

### Anoctamin Superfamily comparisons providing evidence for homology

Pairwise comparisons, using BLASTP [94], were run as a first step in determining the groups and relationships among the Anoctamin superfamily members. These results suggested the groupings into seven distinct families, and the existence of the superfamily. Of all within group BLASTP comparisons, more than 85% attained e-values below10^-10^, while few inter-group comparisons failed to satisfy the e-value cutoff of 10^−3^. By the transitivity principle (if A is homologous to B, and B is homologous to C, then A is homologous to C), these BLASTP inter-family results provide evidence suggesting that all the proteins belong to a single superfamily.

To better support the suggested superfamily, we used our SuperFamily strategy (see Methods). To run these analyses, we selected a negative control set of 87 families containing a total of 3,332 transporter proteins in TCDB with no known relationship with the Anoctamin Superfamily. The first step in the strategy is the expansion of each family by comparison against NCBI’s NR protein sequence database. We ran this step using famXpander (see Methods). Examination of the results from famXpander revealed that members of different families matched the same protein sequences. Common matches were frequent between members of the superfamily (1514 total proteins), while only three common matching proteins (two between TC: 1.A.17.1 and TC: 2.A.1; and one between TC: 1.A.17.1 and TC: 2.A.29) were found against our negative controls. Furthermore, the regions of the common matches covered by the alignments with the members of the different Anoctamin families had overlaps ranging from 300 to 500 aas. In contrast, the regions of the common matches covered by the alignments against the negative controls had overlaps ranging from zero to 40 aas. Therefore, the links between different families of the superfamily, based on the transitivity principle, was strengthened.

**Fig 2:**
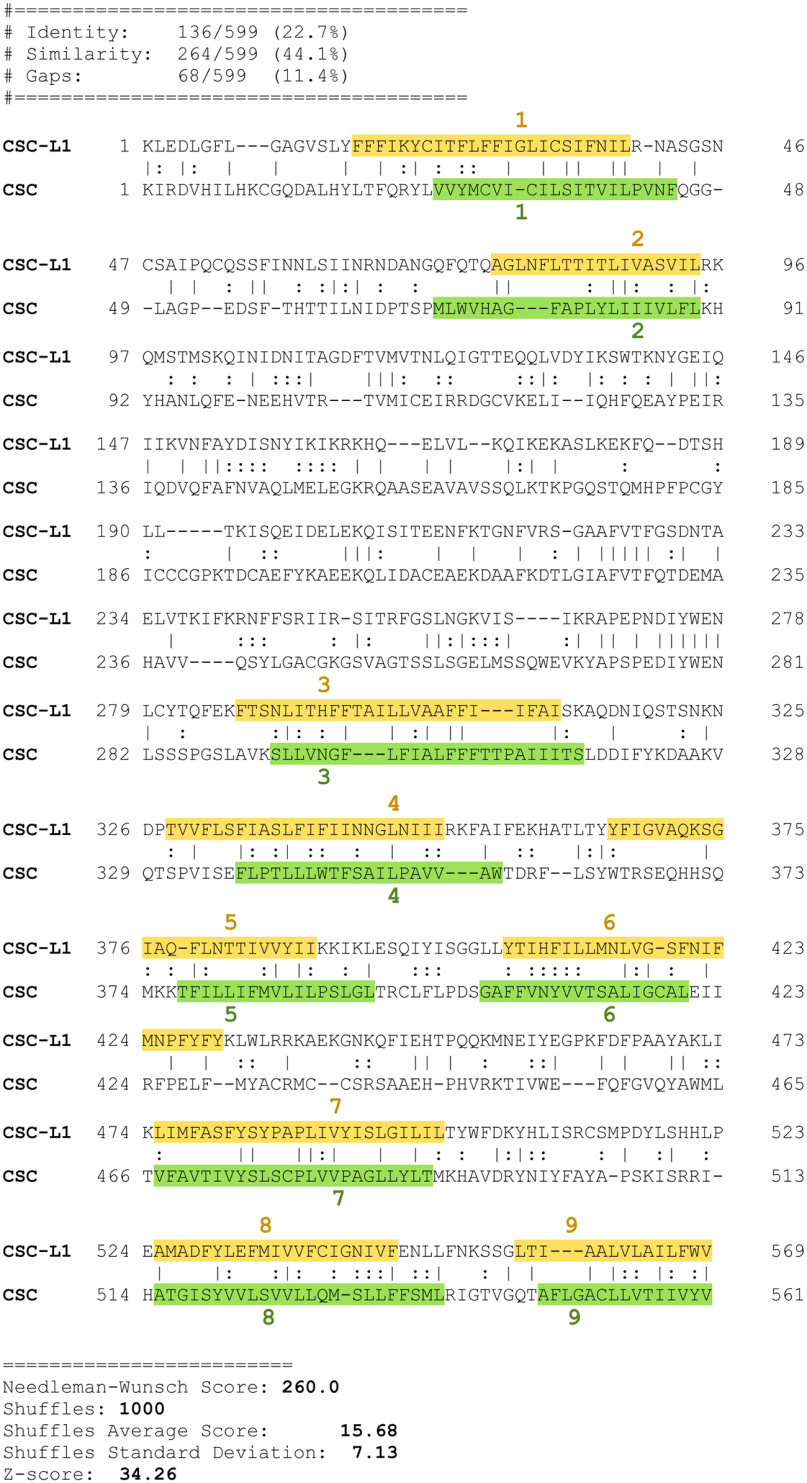
GSAT pairwise alignment of a homolog of the CSC-L1 family (XP_001010624) with a homolog of the CSC family (XP_014661822). The alignment shows the local region identified by Protocol2 that was used as evidence for homology between these two families. Family CSC-L1 has TC: 1.A.17.3 while family CSC has TC: 1.A.17.5. Notice that despite the low identity levels (22.7%), the TMSs align well, and a hydrophilic region between the second and third TMSs is shared (GSAT score 34.2 SD). TMSs were identified by running HMMTOP [96] on the full protein sequences and then mapping the TMS coordinates in the alignment.

To provide further evidence for homology between the families of the Anoctamin Superfamily, GSAT scores between members of the different families were determined [95]. An example of an alignment used as evidence of homology between the CSC and CSC-L1 families is shown in Fig 2. Top scores between families are presented in Table 2. The lowest GSAT score that can be used to relate all seven families was 21.1 SD. Within each of the seven families of the Anoctamin Superfamily.

**Table 2:** Top GSAT scores (expressed in standard deviations (SD)) between members of the seven families in the Anoctamin Superfamily^†^. The inference of homology is based on the Superfamily Principle. See the Methods section for procedural details. The table shows only the highest scores (columns 5–7) that allow the identification of homology transitivity paths A→B→C→D^†^ (columns 1–4) among all seven families. For each row, the cell corresponding to the comparison score in the transitivity path is shaded (lowest score; see columns 5–7). Notice how families in rows 1, 4, 5, 7 and 8 are related by the same protein; that is B = C, which indicates that the same protein has significant alignments with both Family 1 (A; column 1) and Family 2 (D; column 4).

				Comparison Score^†^			
Family 1 (A)	Protein 1 (B)	Protein 2 (C)	Family 2 (D)	A vs B	B vs C	C vs D	Aligned TMSs
1.A.17.1	XP_008873677	XP_008873677	1.A.17.2	104.5	252.9	30.1	8
1.A.17.1	KOO35990	XP_001433607	1.A.17.3	40.2	24.7	201.1	5
1.A.17.2	XP_003294027	XP_001022627	1.A.17.3	177.4	24.4	387.2	6
1.A.17.2	XP_014481354	XP_014481354	1.A.17.4	28.1	401.9	94.8	10
1.A.17.2	XP_014481354	XP_014481354	1.A.17.6	27.6	264.9	26.0	9
1.A.17.3	XP_001010624	XP_014661822	1.A.17.5	72.9	34.3	168.4	9
1.A.17.3	XP_001441614	XP_001441614	1.A.17.7	428.8	226.4	21.1	7
1.A.17.4	EPZ36648	EPZ36648	1.A.17.6	81.3	361.8	42.2	9

^†^ Comparison scores were calculated using the GSAT program with 1000 random shuffles.

Families 1 (A) and 2 (D) are well established family members in TCDB, Protein 1 (B) is homologous to A and Protein 2 (C) is homologous to D. Proteins 1 and 2 were obtained and compared using famXpander and Protocol2, respectively, as described in the Methods section.

To determine whether a 21.1 score was sufficiently high to provide evidence for homology, we compared GSAT scores against numerous negative controls. Homologous proteins in the 87 families used as negative controls were compared with homologues of the ANO family (TC: 1.A.17.1) using the famXpander, Protocol2 and GSAT programs (see Methods). The highest GSAT score obtained for the 87 negative controls was 18.7 SD (S1 Table), with 77 of them having scores ≤ 17 SD. Moreover, the correspondence of TMSs in the sequence alignments against the negative controls did not make sense. For example, the aligned regions included dissimilar numbers of TMSs, and repeat sequences observed for the negative control proteins could not be observed for the Anoctamin Superfamily members. In clear contrast, TMSs aligned well when comparing members of different Anoctamin families.

### Phylogeny of Anoctamin Superfamily members

**Fig 3:**
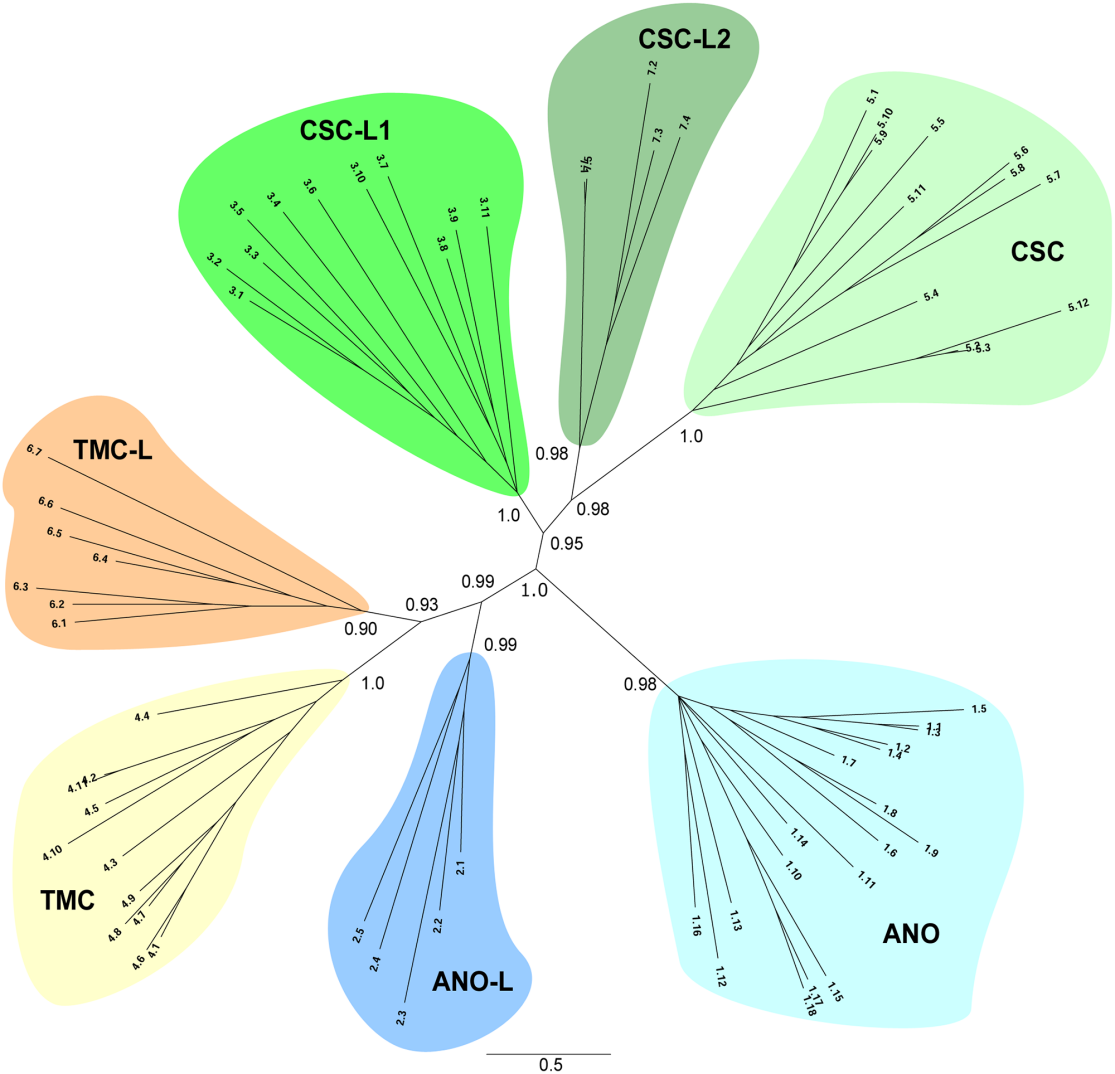
Phylogenetic tree of protein members of the Anoctamin Superfamily. The tree was generated with MrBayes [98]. The multiple alignment used to build this tree was generated with MAFFT [104] and trimmed with trimAL [105] to ensure that each residue position in the alignment contained less than 15% gaps. The seven families are labeled as indicated in the text. The labels of the leaves correspond to the last 2 components of their TC identifier. Complete TC identifiers result from inserting “1.A.17.” to the left of each leaf label.

**Fig 4:**
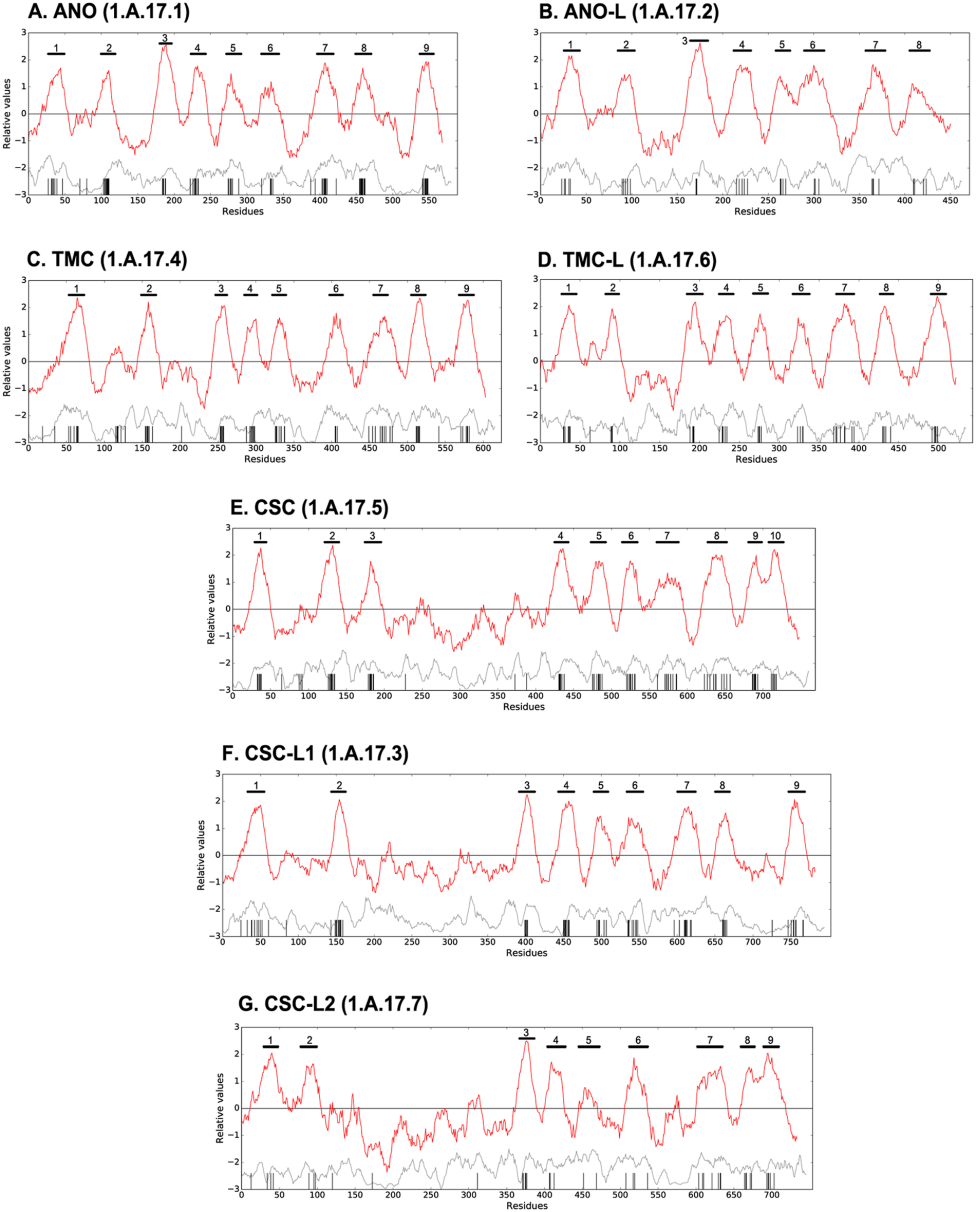
Average topological features of the seven families within the Anoctamin Superfamily. Plots for all families were generated with the AveHAS [106] program. Each plot is composed of two curves. Top dark red lines represent average hydropathy. Bottom gray dotted lines represent average similarity. Predicted TMSs are shown as vertical gray lines. Numbered bars above the hydropathy curves indicate the positions of peaks of hydrophobicity, usually predicted to be TMSs using the HMMTOP [96] and WHAT [95] programs. This figure shows that there are 8 to 10 hydrophobicity peaks in all seven families, which likely correspond to 9 or 10 TMS, since, in this superfamily, some hydrophobicity peaks (such as peak 7 in **A**) are composed of 2 TMSs. The similarity curves indicate that the regions containing TMSs have the highest levels of conservation, and the corresponding multiple alignments shows that they have fewer gaps.

Phylogenetic trees of the expanded Anoctamin Superfamily were constructed using Phylip [97] and MrBayes [98]. In addition, we clustered the sequences based on BLASTP bit scores using SuperfamilyTree [99–102], and based on the Smith-Waterman algorithm as implemented in SSEARCH [103] using our program mkProteinClusters (see Methods). All trees showed the same clustering of sequences, produced essentially the same topology, and, in multiple cases, showed strong statistical support for the nodes separating each family from one another (Fig 3). The only difference was the position of family ANO-L (TC: 1.A.17.2). The clustering generated by SuperfamilyTree (S2 Tree) placed family ANO-L on the same main branch as family ANO (TC: 1.A.17.1). This grouping, together with the average hydropathy and similarity plots (Fig 4) and the conservation of Ca^2+^-binding residues (see section “Analysis of Functional Residues” below), was used to name the family ANO-L. Trees built with MrBayes and Phylip also placed family ANO-L near the center of the tree, but on the same branch and closer to TMC-L (TC: 1.A.17.6), regardless of the fraction of gaps per position allowed per alignment. The program mkProteinClusters arrived at the clustering of families shown in Fig 3 (clustering coefficient of 0.98), although it used bit scores produced by Smith-Waterman alignments to estimate distances (see Methods and S3 Tree).

The newly discovered CSC-L1 and CSC-L2 Families seem to be most closely related to the CSC family, as they form three clearly distinguishable groups on the same branch of the tree. A similar relationship and clustering pattern is observed within the two TMC families (TMC and TMC-L). However, as noted above, the relationship between the ANO and ANO-L families is not as clear, given that ANO-L was found to be located next to ANO (S2 Tree) or next to TMC-L (Fig 3, S1 and S3 Trees) in several trees, with the former also being supported by the conservation of functional residues (see section “Analysis of Functional Residues”) and the latter being supported by their domain organizations (compare Fig 1B and 1D). It is thus apparent that the three major functionally characterized families within the Anoctamin superfamily comprise three principal branches each, with one functionally characterized family (i.e., ANO, TMC, and CSC) per branch.

Because the characterized Anoctamins, TMCs and CSCs, are known to have distinct functions, we suggest that these trees provide guidelines for the functional elucidation of members of the families of unknown function. The four groups of proteins, represented by ANO-L, CSC-L1, CSC-L2, and TMC-L families, were named on the basis of their Pfam domains (Fig 1) and their clustering in the trees (Fig 3 and S1–S3 Trees).

### Topological evaluations

The members of the Anoctamin superfamily were examined and characterized with respect to protein sizes, topologies and organismal phyla (Table 1). All seven families exhibit comparable protein sizes (703–994 aa) and topologies (8–10 hydrophobicity peaks corresponding to 9–10 TMSs), although some are much larger and may consist of “fusion” proteins with additional hydrophilic domains. The spacing of TMSs and the sizes of the loops connecting the TMSs differ significantly. All homologues identified are from eukaryotes, but some families are far more widely distributed than others. For example, members of the ANO-L family are the most restricted in distribution, being found only in animals, while the CSC-L1 family is represented in at least ten phyla. The TMC-L family is not found in animals (Table 1), and CSC-L2 (TC: 1.A.17.7) is found only in unicellular eukaryotes.

**Fig 5:**
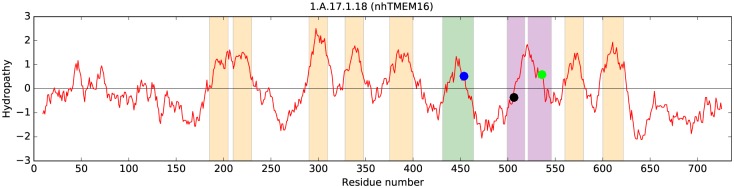
Average hydropathy plot (dark red line) showing the basis for the topological predictions made for the *Nectria haematococca (Fusarium solani)* nhTMEM16 (anoctamin) protein (TC: 1.A.17.1.18), for which x-ray structures are available (PDB IDs 4WIS and 4WIT). Vertical tan bars show the positions of the predicted TMSs using the Loop Finder program (V. S. Reddy and M. H. Saier, unpublished). The green bar shows the position of the α-helix corresponding to TMS 6. This helix was not predicted to be a TMS by this program, HMMTOP [96] or CCTOP [108], although the x-ray structure confirmed that it is one. HMMTOP predicted TMSs 1 and 2 as a single TMS, although the structure confirms that the corresponding hydrophobicity peak is composed of two TMSs. The two purple bars, representing the position of transmembrane helices 7 and 8 in the x-ray structure, were predicted by these programs and AveHAS [106] to be a single TMS (also note the 7^th^ hydrophobicity peak in Fig 4A). This explains the discrepancy in the predictions for different members of the Anoctamin Superfamily (between 8 and 10 TMSs). The locations, in the hydropathy curve, of the three pairs of functional residues that bind Ca^2+^ in TMSs 6, 7 and 8 are depicted with blue, black and green circles, respectively.

Fig 4 shows average hydropathy plots for members of each of the seven families described in Fig 3 and Table 1. These plots depict the average properties as a function of residue position in the multiple alignments created as described in Methods. In each panel, the top dark red lines indicate average hydropathy. Vertical grey bars below the hydropathy/amphipathicity plots represent residues in predicted TMSs by HMMTOP while the dotted gray lines indicate average similarity. High similarity in a hydrophobic region predicted to be a TMS correlates with strong conservation. Well conserved regions with high hydrophobicity (inferred TMSs) are indicated with numbers above hydropathy peaks. A total of 8 to 10 conserved hydrophobicity peaks are identified for each of the seven families, but the actual number of TMSs is likely to be 9 or 10 because some hydrophobicity peaks involve 2 TMSs (Fig 5).

### Lack of recognizable repeats

Attempts were made to identify repeat sequences in members of the Anoctamin Superfamily. However, it was not possible to find significant evidence suggestive of the occurrence of internal sequence repeats using the HHrepID [107] and AncientRep [95] programs. Similarly, examination of the 3-D structure of the fungal homologue, nhTNEM16, failed to reveal the presence of reliable repeat structures. However, if we excluded the loops connecting membrane-spanning α-helices, it was possible to observe a potential 3-TMS structural repeat with borderline significance, RMSD = 3.57 Å over a 60 residue alignment where TMSs 3–5 align with TMSs 6–8 (See Methods and S1 Fig). This value is similar to the RMSD values obtained by comparing known repeat units within members of the MFS (without removing loops and selecting for high coverage alignments). For example, we observed RMSD values of 2.74 Å (over 74 residues) and 3.14 Å (over 95 residues) for three- and four-helix bundles, respectively, for the lactose permease protein (PDB: 2CFP). This is not sufficient evidence to suggest that a sequence duplication event gave rise to the proposed structural repeat. The lack of sequence similarity suggests that either repeat sequences have diverged beyond recognition, or, alternatively, that in contrast to most families of large integral membrane transport proteins [17], members of the Anoctamin Superfamily have not arisen via a route involving intragenic duplication.

### Comparison of predicted TMS topologies with the X-ray structure for the *Nectria haematococca* homologue (TC: 1.A.17.1.18)

As noted above, sequence-based topological predictions (Fig 4) for members of the seven families in the Anoctamin Superfamily showed 8 to 10 hydrophobicity peaks. The 3-d structures of 1.A.17.1.18 (PDB: 4WIS and 4WIT) were therefore compared with the initial 9 TMS topology inferred for this protein. After mapping the inferred TMSs onto the X-ray structure, a general agreement with the organization of α-helices in the membrane plane was observed with the notable exception of the third from the last peak of hydropathy. This broad peak, with a shoulder of hydropathy on the right side, corresponds to two TMSs separated by a β-turn (Fig 5). We suggest that most members of the Anoctamin Superfamily have the 10 TMS topology observed for the *N*. *haematococca* homologue. Proteins in family ANO-L have 8 conserved hydrophobicity peaks (Fig 4); however, as Fig 5 shows, one of these peaks may be composed of 2 TMSs. As discussed below, at least some members of this family may lack the last TMS.

### Analysis of functional residues

**Fig 6:**
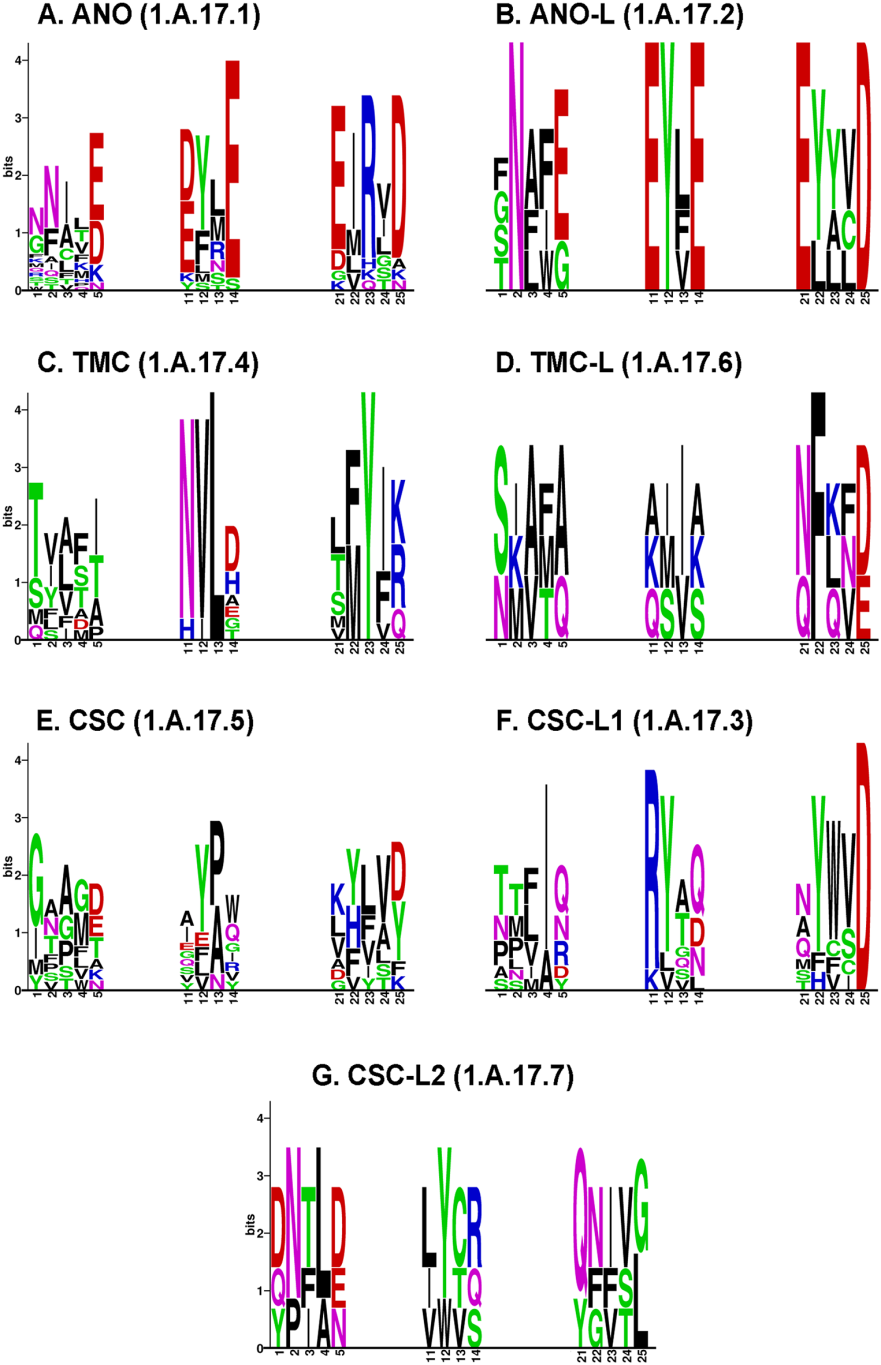
Conservation of functional residues across the Anoctamin Superfamily. The sequence logos illustrate the conservation of the Ca^2+^-binding residues N448, E452, D503, E506, E535 and D539 (columns 1, 5, 11, 14, 21 and 25, respectively) in each family. N448 and E452 are located in TMS 6, D503 and E506 in TMS 7, and E535 and D539 in TMS 8 (Fig 5). Spaces separate residues in the first, second and third Motifs in TMSs 6, 7 and 8, respectively. Positions between pairs of functional residues in the same TMS were included to provide context. Notice that outside families ANO (panel A) and ANO-L (panel B), the residues are poorly conserved, suggesting that different residues are involved in Ca^2+^ binding in the other families.

The 3D structure of the fungal homolog nhTMEM16 [48] contains six functional residues responsible for binding Ca^2+^, which are located in TMS 6 (N448 and E452), TMS 7 (D503 and E506), and TMS 8 (E535 and D539) (Fig 5). We followed two approaches to study the conservation of these and the channel-forming residues for members of the superfamily. First, we generated multiple alignments, combining the proteins of family ANO with the proteins of each one of the other 6 families using MAFFT [104], and compared the positions corresponding to the Ca^2+^-binding residues as well as the TMSs delineating the subunit cavity in nhTMEM16. Second, we used the MEME suite of programs [109] to search for conserved motifs across the superfamily and determined whether identified functional residues are part of the top scoring motifs (Fig 6). For the purpose of the following discussion, the sequences between each pair of Ca^2+^-binding residues in TMSs 6, 7 and 8 will be referred to as Motifs A, B and C, respectively. Families ANO (Fig 6A) and ANO-L (Fig 6B) exhibit the highest level of conservation, with residues, asparaginyl (N), aspartyl (D), and glutamyl (E), predominating in all three of the displayed motifs. The other families show considerable variation, but the observed substitutions frequently involve compatible residues. The TMC family (Fig 6C) shows poor conservation of motif A, while motif B exhibits a largely conserved NVL sequence (columns 11–13), and motif C has a fully conserved Y (column 23). In TMC-L (Fig 6D) the most conserved is motif C, where an NFXXD sequence predominates. In CSC (Fig 6E) no residues predominate. In CSC-L1 (Fig 6F), an I (column 4) predominates in motif A, RY (columns 11 and 12) predominates in motif B and YWVD (columns 22–25) is found in motif C. In CSC-L2 (Fig 6G), no predominant residue is shared with CSC and CSC-L1, except for the Y in column 12 of motif B, and a V in column 24 of motif C.

Focusing on the specific positions of the Ca^2+^-binding residues in the fungal nhTMEM16 protein (Fig 6), only two families, ANO and ANO-L, displayed well conserved D and E residues (Fig 6A and 6B). The rest of the families show considerable variation, but the following positions exhibit compatible substitutions: (1) the N at position 11 in motif B of the TMC family (Fig 6C), (2) the conserved N/Q and D/E at positions 21 and 25 in motif C of the TMC-L family (Fig 6D), (3) the poorly conserved D/E at position 5 in motif A and the D at position 25 in motif C of the CSC family (Fig 6E), (4) the poorly conserved Q/N/D at position 5 of motif A, the Q/D/N at position 14 of motif B and the fully conserved D at position 25 in motif C in family CSC-L1 (Fig 6F), and (5) the D/Q at position 1 and the D/E/N at position 5 of motif A, and the Q at position 21 of motif C in the CSC-L2 family (Fig 6G).

Since several of the known Ca^2+^-binding residues in the fungal nhTMEM16 are not conserved across the superfamily, we sought alternative residues with negative charge or strongly electronegative character that could bind Ca^2+^. This was done by examining residue positions in close proximity in 3D space, one or two helical turns away from the identified Ca^2+^-binding residues shown in Fig 6. That is, residues located about 3.6 or 7.2 residues away from the assigned residues in these transmembrane helical segments. The results were remarkably revealing. S2 Fig illustrates that at these positions (3, 4, 7 or 8 residues from the aforementioned Ca^2+^-binding residues) we found conserved N/D/E/Qs before and/or after the three motifs in all families. The figure also shows the presence of positively charged residues adjacent to (e.g., Motif C, family CSC-L1) or one helical turn away (e.g., Motif C, family ANO) from negatively charged residues. These residues could stabilize the D at the end of motif C. These observations suggest that alternative replacement residues or “helper” residues close to the Ca^2+^-binding residues in nhTMEM16 may participate in Ca^2+^-binding.

**Fig 7:**
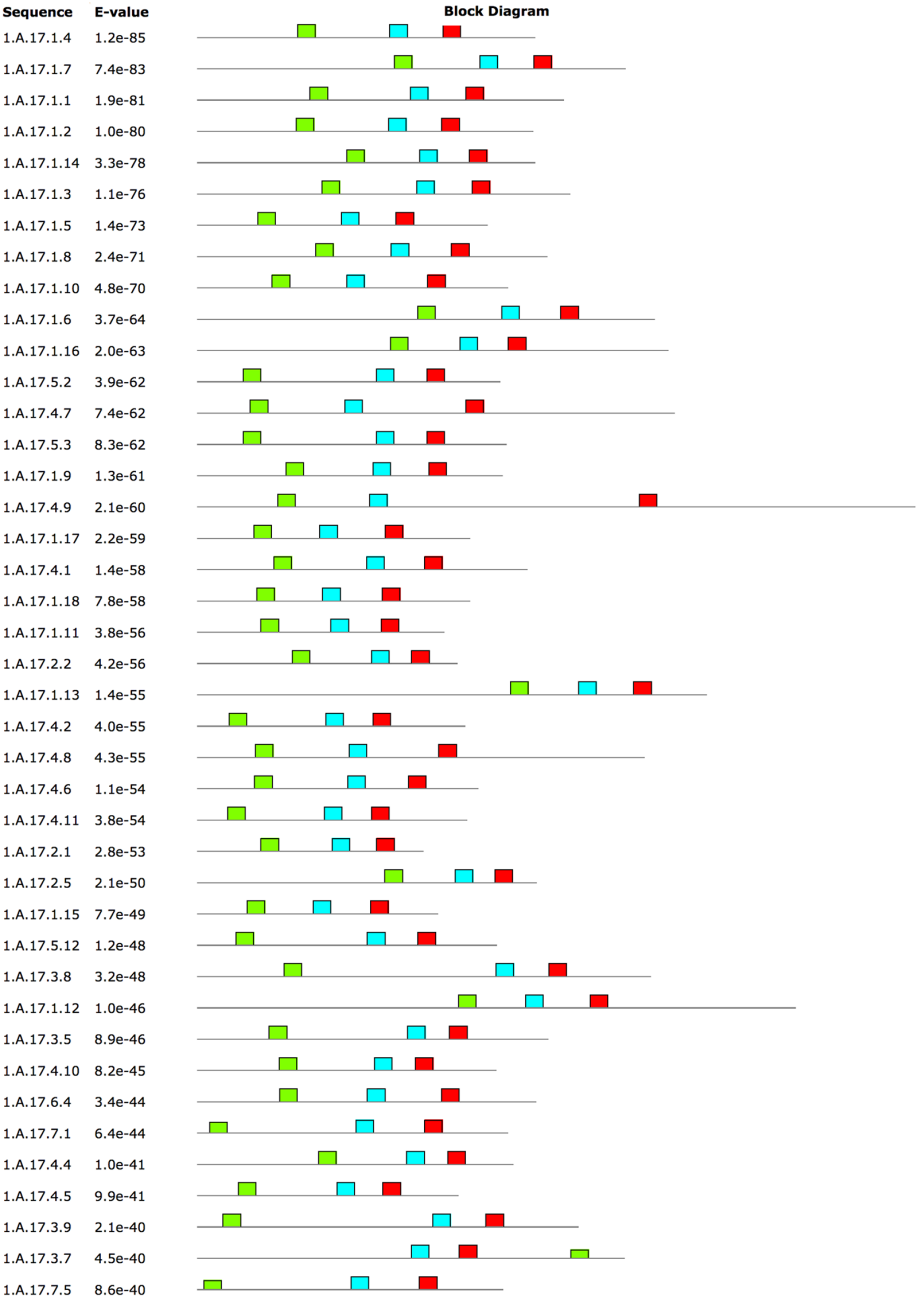
MAST output containing the top 3 motifs identified by MEME. The figure shows sequences with motif E-values < 10^−39^. Motif 1 (red boxes) maps to TMSs 7 and 8, where 4 of the 6 Ca^2+^-binding residues in nhTMEM16 are located. Motif 2 (cyan boxes) maps to TMSs 4 and 5 in nhTMEM16, which form part of the subunit cavity for phospholipid translocation. Motif 3 (green boxes) maps to TMS 1, but this TMS does not interact with Ca^2+^ or the substrate. Our results show that 94% (65/69) of the sequences in the superfamily map Motif 1 to the region that contains 4 of the 6 functional residues that bind Ca^2+^, and 98.5% (68/69) of the sequences map Motif 2 to TMSs 4 and 5.

As discussed above, other positions in the neighborhood of the Ca^2+^-binding residues in nhTMEM16 are well conserved. Thus, we attempted to identify larger conserved motifs across the superfamily. Despite the variation observed in the functional positions, the context provided by the neighboring residues is conserved to the extent that the most significant motif (50 residues long, E-value < 10^−420^) identified by MEME maps precisely to the region containing the functional residues in TMSs 7 and 8 (i.e., D503, E506, E535 and D539) of nhTMEM16 (Fig 7). With the exception of 4 proteins (i.e. 1.A.17.6.1, 1.A.17.6.3, 1.A.17.6.7, and 1.A.17.3.2), for which functional residues could not be properly identified (due to gaps in the corresponding positions or the residues not mapping to the correct hydrophobicity peaks). The location of this motif in all families, as inferred by MAST, maps precisely to the region where the Ca^2+^-binding residues in nhTMEM16 are located. At the superfamily level, the region containing the other two Ca^2+^-binding residues in nhTMEM16, residues N448 and E452 in TMS 6, is poorly conserved outside the ANO family. The second most significant motif (E-value < 10^−335^) maps to TMSs 4 and 5 which are part of the subunit cavity for lipid scrambling in nhTMEM16 and the Cl^-^ channel in mTMEM16A [53]. This motif contains residues E352 and K353 (relative to nhTMEM16), which interact with lipid headgroups and have been associated with robust scrambling [110]. It is clear that these residues do not have the highest levels of conservation (see positions 15–16 in the MEME logo of S3 File). Other charged residues (*e*.*g*. E358 and K373) are much better conserved in this motif. Notwithstanding the poor conservation of some residues, with one exception (1.A.17.6.1), all proteins in the superfamily mapped this motif to the regions identified to be homologous to TMSs 4 and 5 in nhTMEM16 (see Methods). The third most significant motif maps to TMS 1 in nhTMEM16, but this TMS is not involved in binding Ca^2+^, nor is known to interact with the substrate. In 2009, Hahn *et al* [78] identified regions containing these 3 motifs (relative to nhTMEM16 TMS1, TMS 4–5 and TMS 7–8) between the ANO and TMC families. In their alignments, albeit unknown at that time, the residues that bind Ca^2+^ in ANO are not highly conserved within the region. Other residues in TMSs 7–8 (i.e., the sequence PL[A/L]P) are clearly better conserved in these two families (ANO and TMC). This is in agreement with our observation of poor conservation of Ca^2+^-binding residues (Fig 6C and 6D). Our analyses also show that these 3 motifs, are well conserved across all seven families within the superfamily. S3 File contains the output of MEME and MAST applied to the whole superfamily.

## Discussion

In this report, we provide bioinformatic evidence that strongly suggests that the Anoctamin family of channel proteins (ANO) is related to both the TMC and CSC families. These three families are now grouped into a larger superfamily which we have called the Anoctamin Superfamily. In addition to these three families, we have found four novel families of unknown function that belong to the superfamily. We named them the Anoctamin-like (ANO-L), CSC-like (CSC-L1 and CSC-L2), and TMC-like (TMC-L) families based on their clustering patterns (Fig 3 and S1–S3 Trees). Thus, we have expanded the Anoctamin Superfamily, from 3 to 7 families. The diverse functions of members of the former three families in cation, anion and lipid transport suggest that the proteins of unknown function will similarly exhibit diverse functions, perhaps more divergent than those currently recognized. We nevertheless anticipate closer functional overlap between TMC and TMC-L, as well as between CSC and both CSC-L1 and CSC-L2. ANO-L could be closer in function to either ANO or TMC. Our analyses of both the TMS and tree topologies of the proteins in all of these families suggest that they are all similar in their basic domain architectures (Fig 1), although they cluster as seven distinct families on the trees (Fig 3). These observations should be useful guides for future studies.

Our protein sequence analyses identified 8 to 10 conserved hydrophobicity peaks (Fig 4) that likely correspond to 9 or 10 TMSs, based on the observation that one hydrophobicity peak can sometimes correspond to 2 TMSs (Fig 5). The predicted 8 and 9 TMS topology conflicts with the high resolution fungal Anoctamin structure, which shows a 10 TMS topology [20, 48]. Based on the known structure and the topological analyses reported here, we suggest that most superfamily members have a 10 TMSs topology. Although members of the ANO-L family appear to have 9 TMSs, having lost the C-terminal TMS.

Since there is a notable difference between the substrates of Anoctamins, TMCs and CSCs (e.g., anions *vs*. cations, in addition to lipids), we suspect that the mechanisms of channel activation will prove to be the most strongly conserved features of this superfamily, as supported by our analysis of the conservation of sequence motifs that include known Ca^2+^-binding and channel-forming functional residues. However, it is noteworthy that only two of the families (ANO and ANO-L) show conservation of the Ca^2+^-binding residues known for nhTMEM16. We suggest that the mechanism(s) of translocation and regulation mediated by these proteins differ in detail for members of the dissimilar families. Proposals as to the mechanisms of lipid flipping by some members of the superfamily have recently been considered by Brunner et al. [20, 48], as well as by Whitlock and Hartzell [75].

When using homology-based approaches to identify potential drug targets, it may be equally important to consider transport mechanisms and substrate selectivities. Understanding which domains of each protein share recognizable homology should allow researchers to dissect the subfunctions of these proteins and design therapies to target proteins that are important in disease progression. However, only high-resolution X-ray structures, such as those published by Brunner *et al*. 2014 [48] (see also [20, 22]) coupled with detailed biochemical and genetic analyses, are likely to resolve the controversies regarding the detailed functions, mechanisms and regulatory features of these proteins.

## Methods

### Examining conserved domains within members of the Anoctamin Superfamily

All members of the ANO, TMC, and CSC families recorded in TCDB were used as query sequences for searches against the Pfam [91] and NCBI’s Conserved Domain Database, CDD [93, 111]. Pfam scans were run using hmmscan, from the HMMer software suite [112] using a gathering threshold. If a family member did not return a significant hit with the most frequent Pfam domain observed for that family (present in at least 50% of the members), then the matching sequence regions of the family members that did report a hit were collected and aligned with the Smith-Waterman algorithm, as implemented in SSEARCH36 [103], to the sequence where the domain was not identified. If a significant alignment was found (E-value < 10^−3^; with at least 70% overlap of the domain sequences), then the domain was regarded as present or “rescued” in the protein without an initial Pfam hit.

CDD scans were run using rpsblast from NCBI’s blast suite [94]. The options for running rpsblast were an e-value threshold of 10^−2^ (as recommended by the authors), compositional-based statistics, and soft-masking of low-information segments.

### Clustering and phylogenetic analyses

To investigate the relative divergence of each family inside the Anoctamin superfamily, we used several methodologies to generate clusters and phylogenetic trees. Proteins listed under TC: 1.A.17 (see S1 File) were thus grouped using the programs mkProteinClusters (https://github.com/SaierLaboratory/TCDBtools), SuperfamilyTree [99–102], Phylip [97] and MrBayes [98]. Multiple alignments were generated with MAFFT [104] using the L-INS-i method (see S2 File). Poorly aligned positions with gaps were removed using trimAL [105]. For each multiple alignment, 3 trimmed alignments were built by keeping positions with gap maxima ranging from 15% to 25%, with increments of 5%. Alignments with fewer gaps were not considered to prevent the alignments from becoming too short. The program mkProteinClusters runs hierarchical clustering as implemented in the R package (https://www.R-project.org/) on a distance matrix calculated from bit scores produced from local protein alignments within the superfamily performed with BLASTP [113], FASTA36 [103], SSEARCH [103], or UBLAST [114]. Clusters were produced using the Ward agglomerative method. SuperfamilyTree uses tens of thousands of BLAST bit scores to derive 100 sampled trees [99–102]. These trees were then averaged into a consensus tree using FITCH and CONSENSE from the Phylip software suite with default parameters. Phylogenetic trees created using the Phylip suite were built using the programs NEIGHBOR, FITCH and PROML with 100 bootstrap replicas. MrBayes was used to generate trees assuming that substitution rates per position are different and follow a gamma distribution with 4 rate categories. Posterior probabilities were estimated using Metropolis coupling (1 cold and 3 heated chains) and at least 600,000 generations or until the average standard deviation of split frequencies fell below 0.01. Trees were drawn with FigTree (http://tree.bio.ed.ac.uk/software/figtree/) and the Interactive Tree of Life (iTOL: http://itol.embl.de/) [115]. To increase clarity, the tree in Fig 3 displays only the bootstrap support values of the main nodes separating the families. However, the original tree used to generate Fig 3 is provided in the Supporting Information section (S1 Tree).

### Negative control set for homology

It is well documented that transmembrane segments contain low complexity hydrophobic regions that may generate statistically significant sequence similarity. However, that does not necessarily suggest shared ancestry, as it may instead be the result of common selective pressures due to physical-chemical constraints in the membrane environment [116]. Our strategy to overcome this hurdle consists in comparing the GSAT [95] scores among sets of potentially related transporters to the scores obtained in alignments between transporters thought to be unrelated. GSAT computes a z-score that compares the alignment score of two real biological sequences to the average score obtained within a sample of alignments of shuffled sequences. In this context, the alignment scores of randomized sequences are not used as a null model to directly infer the significance of an alignment (e.g., a p-value). Instead, the z-score provides a scale or baseline that can be used to rank alignment scores of homologous and non-homologous transporters. The goal is to identify a critical value for the z-score that discriminates between homologous and non-homologous relationships for the families included in the positive and negative controls. We selected a set of 87 families from TCDB with no known relationship to the Anoctamin superfamily as negative controls. The 3,332 sequences within this negative control set were compared against members of the ANO family (TC: 1.A.17.1) in the same way used to compare the members of the superfamily with each other (see next section).

### Identifying homology between clusters generated by the phylogenetic trees

We wrote the script, “areFamiliesHomologous”, to automate the three main steps in our strategy to infer homology between families of transporters based on the transitivity principle [102]. This pipeline connects multiple programs, including those in the BioV suite (https://github.com/SaierLaboratory/BioVx) [95], to make the process significantly faster, more comprehensive, and to eliminate the possibility of human errors.

First, we made an exhaustive search for candidate homologous proteins in each cluster of the phylogenetic tree with our program famXpander, which starts by running local BLAST [113] searches against the NCBI non-redundant (NR) database. Alignments had to cover at least 45% of the query and yield an E-value < 10^−2^. Then famXpander extracted the sequences of the aligned regions and removed redundancies with CD-HIT [117] using a 90% identity threshold. Finally, famXpander created a file of non-redundant putatively homologous sequences in FASTA format.

Second, Protocol2 from the BioV Suite [95] of programs was used to find similarities between pairs of lists of putative homologues obtained by famXpander. This program generates local pairwise alignments with the exhaustive Smith-Waterman algorithm, as implemented in SSEARCH from the FASTA suite of programs [103], for all possible pairs of proteins between two lists of homologues and estimates an initial GSAT score based on 500 shuffles. For each pairwise alignment, Protocol2 shows labeled TMSs in each sequence as predicted by HMMTOP [96]. These are then verified with hydropathy plots to identify which TMSs are conserved between two families of transporters.

Third, the top scoring alignments, showing at least 5 overlapping TMSs and a minimal alignment length of 150 residues, were verified using GSAT with 1000 shuffles. GSAT z-scores were calculated for i) candidate homologues between different families, and ii) the original transport protein in TCDB (i.e. the query sequence for famXpander) and its corresponding BLAST match. Before calculating final GSAT scores, we inspected the alignments to make sure that only sections containing hydrophobicity peaks were included; hydrophilic segments at either the N- or the C-terminus were removed. If we labeled two proteins in different TCDB families as A and D, the BLAST hits of A as B and the hits of D as C, then we could calculate the GSAT scores for A-B, B-C, and C-D. The lowest of the three scores was regarded as the comparison score. The three scores are given in Table 2, but only the comparison scores are presented in S1 Table.

### Multiple alignments of homologues and average hydropathy/amphipathicity/similarity plots

Using the algorithm L-INS-i as implemented in MAFFT [104], a multiple alignment for each family was created. To prevent non-conserved regions from showing in the hydropathy plots, we required that at least 30% of the proteins in a family must contribute residues to any position in the alignment. Thus, we used trimAL [105] to remove positions with >30% gaps. Average hydropathy plots were then created with the web-based program AveHAS (Average Hydropathy, Amphipathicity and Similarity; http://biotools.tcdb.org/baravehas.html) [106] using these multiple alignments. To improve clarity, only the hydropathy curves are shown, and any conserved hydrophilic regions at either the N- or the C-terminus were removed in order to focus the alignment on the transmembrane domains. AveHAS plots were used to study the conservation of TMSs at the family level.

### Identification of internal sequence repeats

HHrepID [107] and AncientRep [95] were used with default settings to seek possible internal repeats (duplications) within each family of proteins. HHrepID uses a single protein sequence to locate potential occurrences of internal duplications by using HMM-HMM comparisons. AncientRep uses a multiple alignment as input and allows the user to select regions in the alignment based on AveHAS [106] plots to guide the search of repeats. GSAT scores between two sections of the alignment are generated. No significant repeats were identified in any member of the Anoctamin Superfamily using these approaches.

### Search of structural repeats within the 3D-structure of 1.A.17.1.18

The membrane-spanning α-helices in structures 4WIS and 4WIT were cut in sets of 3, 4 and 5 helix bundles. All non-overlapping helix bundles of the same size were aligned with the CCP4 [118] implementation of the SSM superpose algorithm [119]. No significant alignments with RMSD values of < 4 Å with coverage of at least 60 residues were obtained. As a second approach, we considered excluding the loop regions connecting α-helices within the bundles to compare only the position and orientation of the TMSs. We identified two adjacent three-helix bundles containing TMSs 3–5 and 6–8 that produced an RMSD = 3.57 Å with an alignment of 60 residues (see S1 Fig). If loops were considered, this alignment was also observed with a significantly higher RMSD value (4.68 Å over 79 aligned residues).

### Identification of distant family members within the Anoctamin Superfamily

All sequences from a reference family in TCDB were automatically extracted with the program extractFamily, which connects to TCDB, downloads the sequences and returns them in one of several formats (i.e. fasta, column or blast database). Then, famXpander is run on all proteins of the reference family using BLASTP searches against the NCBI non-redundant protein database in order to get a list of non-redundant BLAST hits showing a minimal alignment coverage (e.g. 70% of the query sequence) and an E-value < 10^−2^. Next, we ran our program findDistantFamilyHomologs that searches for distant members of any given family of transporters. The program first parses the output of famXpander and discards all hits with E-values below a predefined threshold value (e.g., 10^−5^) as they are already represented in TCDB. HMMTOP is then run on the sequences of the remaining BLAST hits with higher E-values, and only sequences with a user-defined minimal number of predicted TMSs are further considered. The remaining sequences are then BLASTed against TCDB to produce a set of proteins that do not have a more significant hit with a family other than the query family, and the e-value is not lower than a predefined threshold. The program then removes redundant sequences from the resulting list of candidate homologs based on a given E-value threshold (e.g., <10^−5^), although redundancy is allowed if their sequence length ratio is large (e.g., >1.8). It reports the accession numbers, preferably UniProt IDs if available, of the resulting distant candidate homologs. This list is finally manually curated to select for the most likely true distant members of the query family.

### Conservation of functional residues

Seven multiple alignments were generated using the algorithm L-INS-i as implemented in MAFFT [104]. The first alignment includes only the members of the ANO Family; the other six alignments correspond to the combination of the proteins in the ANO Family with the proteins in each one of the other 6 families. Columns corresponding to the Ca^2+^-binding residues and the subunit cavity in the structure of nhTMEM16 were identified. S3 Fig shows one representative sequence from each family, illustrating the positions of the Ca^2+^-binding residues. Notice that these residues are located in the fourth to last (TMS 6) and third to last (TMS 7–8) hydrophobicity peaks. The only exception is family ANO-L, where the multiple alignment suggested that all 5 members lack the last hydrophobicity peak (TMS 10 in nhTMEM16; Fig 4 and S3B Fig), even when the actual functional residues are highly conserved (Fig 6B). This is supported both by the position of the functional residues and by high GSAT scores in Protocol2 alignments between members of the ANO and ANO-L families, where the alignments do not include the characteristic 10^th^ TMS of the ANO family (data not shown). Sequences with gaps in the positions of functional residues were also removed. A total of ten sequences (14%) were not considered for the study of conservation of Ca^2+^-binding residues, due to the uncertainty associated with their locations, leaving family ANO with 18 members, Family ANO-L with 4, Family TMC with 10, Family TMC-L with 3, Family CSC with 10, Family CSC-L1 with 10 and Family CSC-L2 with 4 members. S4 Fig shows three examples of sequences that were disregarded because they did not behave as the rest of the members in the superfamily (see S3 Fig). Sequence logo plots were generated with the program SEQLOGO [120] for all filtered alignments focusing on the positions of the functional residues (Fig 6).

The full sequences of all proteins in the superfamily that passed our filtering criteria were used to run MEME [109] in order to search for the top 5 motifs of length 20 to 60 aas (with 5-residue increments and E-value < 10^−100^). We used a maximum of 1000 iterations and a minimal distance of 10^−7^ between motif frequency matrices to achieve convergence. We worked with motifs of 50 residues because this motif length included most of the Ca^2+^-binding residues in the nhTEM16 structure. Of the top 5 motifs we searched, only three had a MEME E-value < 10^−100^. We used the motifs discovered by MEME to run MAST and locate the motifs (E-value < 10^−5^) in all proteins within the superfamily. Relative to the structure of nhTMEM16, motif 1 maps to the region containing 4 of the 6 residues that bind Ca^2+^, motif 2 maps to TMSs 4–5, which form part of the subunit cavity, and motif 3 maps to TMS 1. See text for discussion of the results. S3 File shows the results of MEME and MAST applied to the Anoctamin Superfamily.

## Supporting information

S1 Table Top GSAT scores of the ANO family (1.A.17.1) versus all 87 families in the negative control. The comparisons between each pair of families was carried out using famXpander, Protocol2 and GSAT as specified in Methods. Scores below 15 were deemed as sufficiently low to obviate the need of further analysis. Scores above 15 were subject to the same analysis used to generate Table 2 in the manuscript, but the table shows only the comparison score. That is, the lowest of the three scores A-B, B-C, and C-D (see main text and Table 2). As described in the text, high scoring families in the negative control did not show TMS alignments that made evolutionary sense. GSAT scores ≥ 17 are shaded. For convenience, this table is also provided in CSV format as file: S1_table.csv.
(CSV)

S1 Fig Searching for structural repeats. The membrane spanning α-helices in the structures of the fungal homologue (TC: 1.A.17.1.18; PDB: 4WIS and 4WIT) were cut in sets of non-overlapping three-helix bundles. Bundles were then aligned using the rigid SUPERPOSE algorithm as detailed in Methods. The top scoring alignments of helix bundles containing TMSs 3–5 (light yellow color) and 6–8 (dark brown color) are shown using two approaches. Labeled arrows identify each pair of aligned helices. **A.** Front view of the direct alignment of bundles (RMSD = 4.68Å over 79 residues). **B**. Bottom view of the alignment in A. **C.** Front view of the alignment when loops connecting helices are excluded (RMSD = 3.57Å) over 60 residues. **D.** Bottom view of the alignment in C. The noticeable improvement in the alignment RMSD, when comparing A and C, shows that despite the variability in loop regions, the actual TMSs have similar organization in three-dimensional space.
(TIF)

S2 Fig Illustration of residues D/E/N/Q/K/R/S in positions preceding and following the motifs A, B, and C. The residues within these 3 motifs are highlighted, and the aforementioned residues outside of these motifs are shown to illustrate the possible alternative residues that might function in Ca^2+^ binding (see Fig 6 and Discussion in text). Numbers preceding and following motif labels represent the position away from these motifs. A dash represents a residue not cited above. The first and last positions of each motif correspond to the Ca^2+^-binding residues in TMS 6, 7 and 8, respectively, of the nhTMEM16 homolog. Motifs were found as described in Methods.
(PDF)

S3 Fig Hydropathy plots illustrating the positions and conservation of functional residues in representative proteins of each family within the Anoctamin Superfamily. The locations of the Ca^2+^-binding residues in TMS 6 (blue circles), TMS 7 (black circles) and TMS 8 (green circles) are shown relative to nhTMEM16. Positions of the transmembrane α-helices (tan bars) in nhTMEM16 (1.A.17.1.18) are drawn as observed in the corresponding 3D-structure (**A**). Tan bars in the rest of the panels (**B**-**G**) indicate hydropathy peaks. Notice how the functional residues in family ANO (**A)** are located in the fourth to last (TMS 6) and third to last peaks (TMSs 7–8) of hydrophobicity. This is true for all families, except ANO-L (**B)** where they are located in the third to last and second to last peaks of hydrophobicity. This suggests that the last hydrophobicity peak (TMS 10 in ANO) is missing from **B**. All five members of family ANO-L (1.A.17.2) show the same pattern (see Discussion in text), except for member 1.A.17.2.3 which also lacks TMS 9 (see S4A Fig).
(PDF)

S4 Fig Hydropathy plots of proteins that were not considered for the analysis of conservation of Ca^2+^-binding residues. The locations of the Ca^2+^-binding residues in TMS 6 (blue circles), TMS 7 (black circles) and TMS 8 (green circles) are shown relative to nhTMEM16. Tan bars illustrate the locations of hydrophobicity peaks. **A.** Protein from ANO-L (1.A.17.2.1) is missing the last two hydrophobicity peaks corresponding to TMSs 9 and 10 in nhTMEM16. This is suggested because the functional residues are in the right locations relative to the TMS where they were found and because alignments with members of the ANO family do not include the last 2 TMSs (data not shown). **B.** A protein from TMC-L (1.A.17.6.2) is missing the last hydrophobicity peak (S3A and S3D Fig). **C**. A protein from CSC-L1 (1.A.17.3.2) maps the functional residues in TMS 7–8 to a non-hydrophobic region that includes gaps in positions associated with Ca^2+^-binding residues. All proteins are, nevertheless, true members of their respective families because they all contain the relevant Pfam domains (Fig 1 in the text), produce high GSAT scores in Protocol2 comparisons (see Methods in text), and recover other members of their own family when blasted against TCDB.
(PDF)

S1 Tree Original tree file used to generate Fig 3. This tree was generated using the MAFFT program as described in Methods. Notice how family ANO-L is located on the same branch as family TMC and TMC-L. This file can be easily opened with any tree viewing application (e.g. FigTree).
(TREE)

S2 Tree Tree generated with the SuperfamilyTree program. This tree is very similar to the S1 Tree, except that it groups family ANO-L on the same branch as family ANO. This file can be easily opened with any tree viewing application (e.g. FigTree).
(TREE)

S3 Tree Tree generated with the mkProteinClusters program. This tree generates the same family groupings as does the S1 Tree. This file can be easily opened with any tree viewing application (e.g. FigTree).
(TREE)

S1 File All sequences in the Anoctamin superfamily that were considered in this report. Sequences are provided in FASTA format.
(FAA)

S2 File Multiple alignment used to generate the tree in Fig 3 in the manuscript. The alignment was generated with the L-INS-i algorithm as implemented in MAFFT and trimmed with the trimAL program to keep positions with less than 15% gaps (See Methods). Alignment is provided in FASTA format.
(FAA)

S3 File Conserved motifs in the Anoctamin Superfamily. The file contains the output of MEME and MAST for the entire Anoctamin Superfamily.
(ZIP)
