# Supplementary figures and images for: Bioinformatic characterization of the Anoctamin Superfamily of Ca2+-activated ion channels and lipid scramblases
Source: PLoS One. 2018 Mar 26;13(3):e0192851. doi: 10.1371/journal.pone.0192851 (PMC5868767; doi:10.1371/journal.pone.0192851)

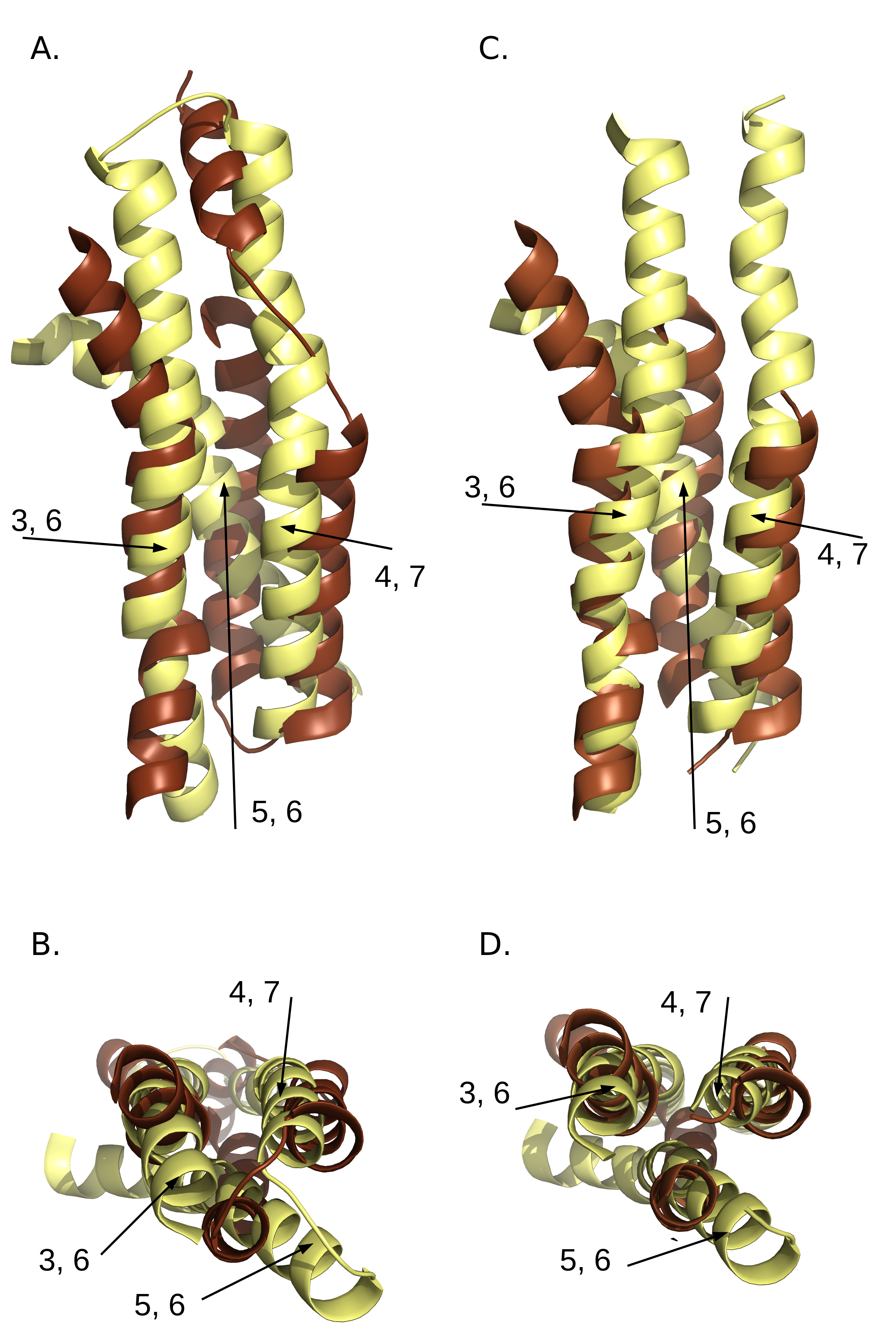

Supplement: S1 Fig — The membrane spanning α-helices in the structures of the fungal homologue (TC: 1.A.17.1.18; PDB: 4WIS and 4WIT) were cut in sets of non-overlapping three-helix bundles. Bundles were then aligned using the rigid SUPERPOSE algorithm as detailed in Methods. The top scoring alignments of helix bundles containing TMSs 3–5 (light yellow color) and 6–8 (dark brown color) are shown using two approaches. Labeled arrows identify each pair of aligned helices. A. Front view of the direct alignment of bundles (RMSD = 4.68Å over 79 residues). B. Bottom view of the alignment in A. C. Front view of the alignment when loops connecting helices are excluded (RMSD = 3.57Å) over 60 residues. D. Bottom view of the alignment in C. The noticeable improvement in the alignment RMSD, when comparing A and C, shows that despite the variability in loop regions, the actual TMSs have similar organization in three-dimensional space. (TIF) [file pone.0192851.s002.tif]

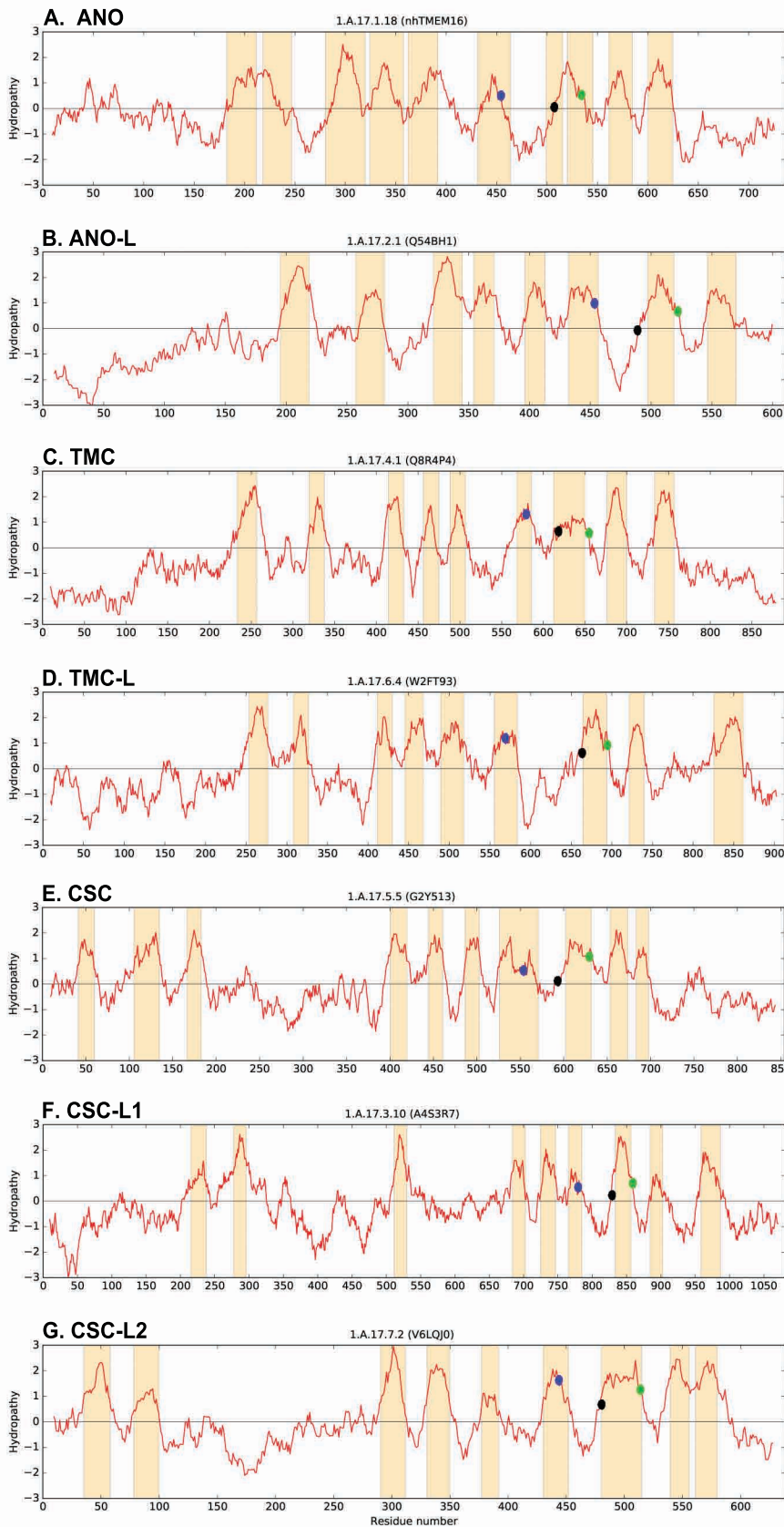

Supplement: S3 Fig — The locations of the Ca2+-binding residues in TMS 6 (blue circles), TMS 7 (black circles) and TMS 8 (green circles) are shown relative to nhTMEM16. Positions of the transmembrane α-helices (tan bars) in nhTMEM16 (1.A.17.1.18) are drawn as observed in the corresponding 3D-structure (A). Tan bars in the rest of the panels (B-G) indicate hydropathy peaks. Notice how the functional residues in family ANO (A) are located in the fourth to last (TMS 6) and third to last peaks (TMSs 7–8) of hydrophobicity. This is true for all families, except ANO-L (B) where they are located in the third to last and second to last peaks of hydrophobicity. This suggests that the last hydrophobicity peak (TMS 10 in ANO) is missing from B. All five members of family ANO-L (1.A.17.2) show the same pattern (see Discussion in text), except for member 1.A.17.2.3 which also lacks TMS 9 (see S4A Fig). (PDF) [file pone.0192851.s004.pdf]

### A. ANO-L

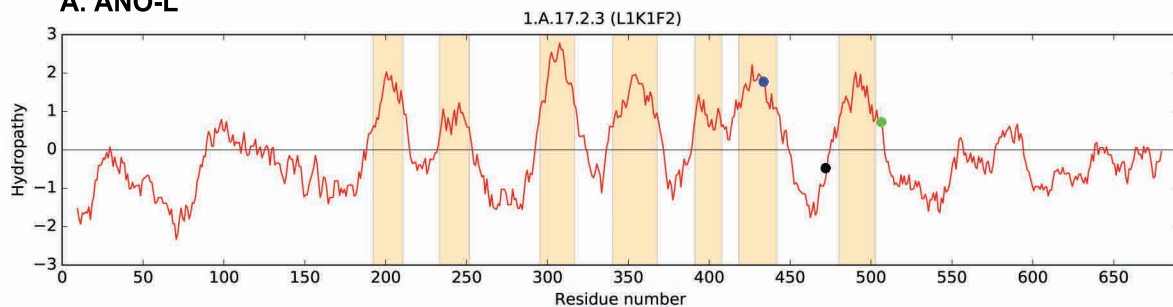

### B. TMC-L

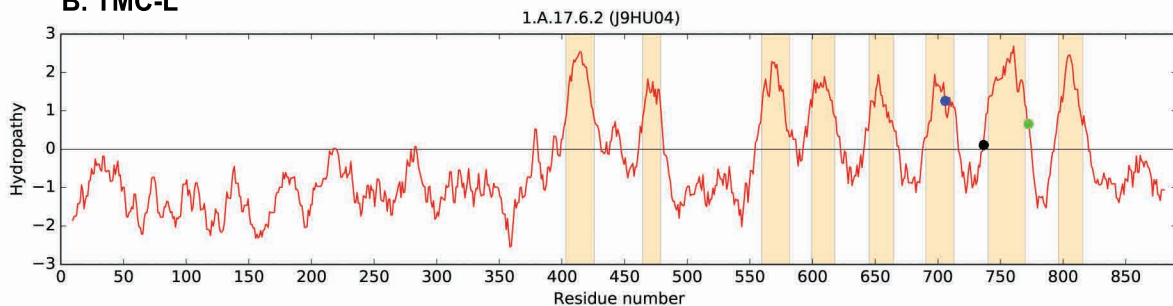

### C. CSC-L1

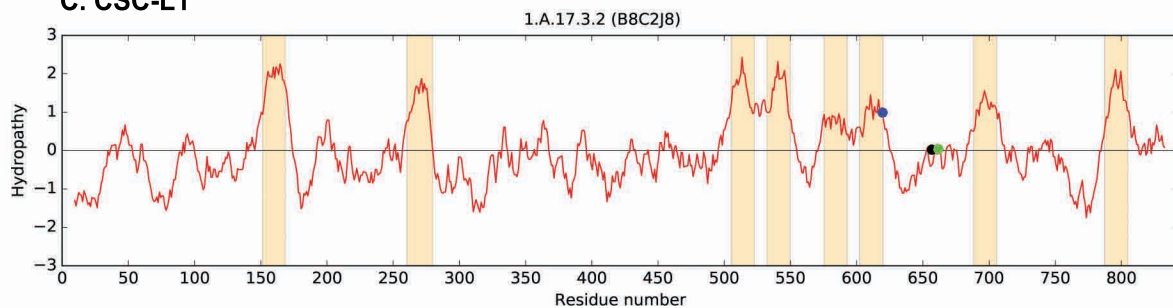

Supplement: S4 Fig — The locations of the Ca2+-binding residues in TMS 6 (blue circles), TMS 7 (black circles) and TMS 8 (green circles) are shown relative to nhTMEM16. Tan bars illustrate the locations of hydrophobicity peaks. A. Protein from ANO-L (1.A.17.2.1) is missing the last two hydrophobicity peaks corresponding to TMSs 9 and 10 in nhTMEM16. This is suggested because the functional residues are in the right locations relative to the TMS where they were found and because alignments with members of the ANO family do not include the last 2 TMSs (data not shown). B. A protein from TMC-L (1.A.17.6.2) is missing the last hydrophobicity peak (S3A and S3D Fig). C. A protein from CSC-L1 (1.A.17.3.2) maps the functional residues in TMS 7–8 to a non-hydrophobic region that includes gaps in positions associated with Ca2+-binding residues. All proteins are, nevertheless, true members of their respective families because they all contain the relevant Pfam domains (Fig 1 in the text), produce high GSAT scores in Protocol2 comparisons (see Methods in text), and recover other members of their own family when blasted against TCDB. (PDF) [file pone.0192851.s005.pdf]

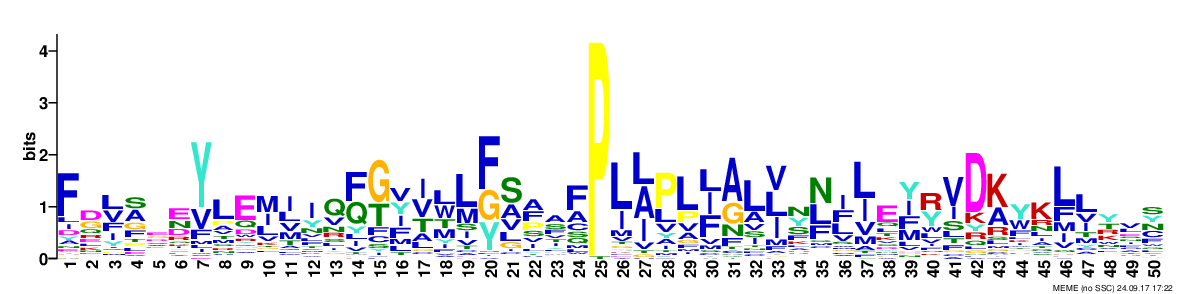

Supplement: S3 File — The file contains the output of MEME and MAST for the entire Anoctamin Superfamily. (ZIP) [file pone.0192851.s011.zip › meme_5m_w50/logo1.png]

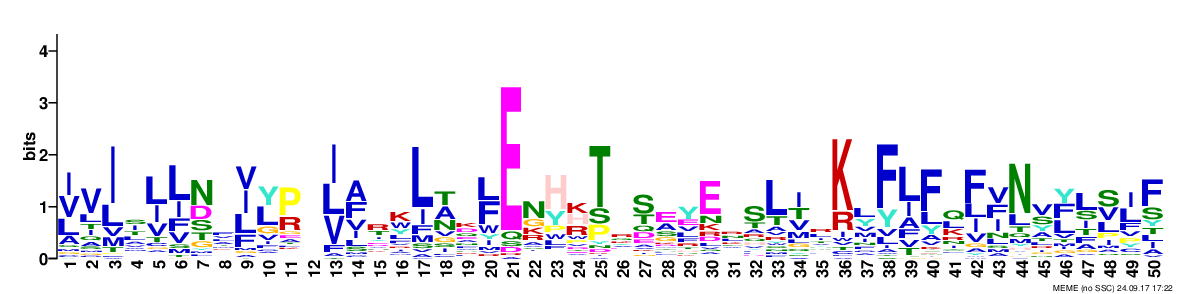

Supplement: S3 File — The file contains the output of MEME and MAST for the entire Anoctamin Superfamily. (ZIP) [file pone.0192851.s011.zip › meme_5m_w50/logo2.png]

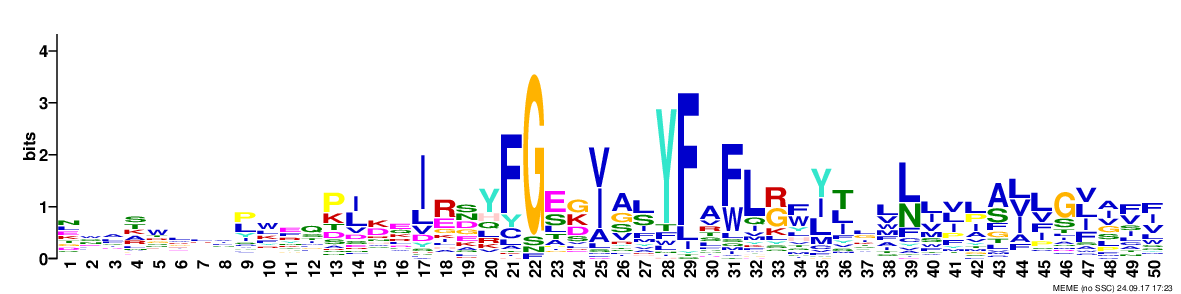

Supplement: S3 File — The file contains the output of MEME and MAST for the entire Anoctamin Superfamily. (ZIP) [file pone.0192851.s011.zip › meme_5m_w50/logo3.png]

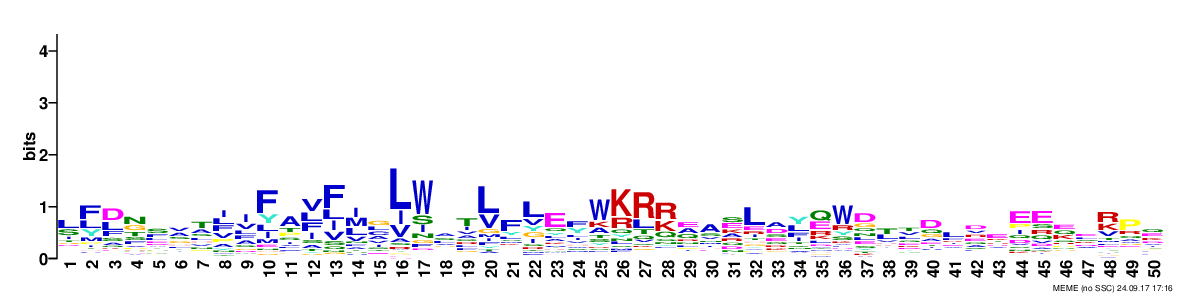

Supplement: S3 File — The file contains the output of MEME and MAST for the entire Anoctamin Superfamily. (ZIP) [file pone.0192851.s011.zip › meme_5m_w50/logo4.png]
