# Supplementary material for: Bioinformatic characterization of the Anoctamin Superfamily of Ca2+-activated ion channels and lipid scramblases
Source: PLoS One. 2018 Mar 26;13(3):e0192851. doi: 10.1371/journal.pone.0192851 (PMC5868767; doi:10.1371/journal.pone.0192851)
Supplement: S2 Fig — The residues within these 3 motifs are highlighted, and the aforementioned residues outside of these motifs are shown to illustrate the possible alternative residues that might function in Ca2+ binding (see Fig 6 and Discussion in text). Numbers preceding and following motif labels represent the position away from these motifs. A dash represents a residue not cited above. The first and last positions of each motif correspond to the Ca2+-binding residues in TMS 6, 7 and 8, respectively, of the nhTMEM16 homolog. Motifs were found as described in Methods. (PDF) [file pone.0192851.s003.pdf]

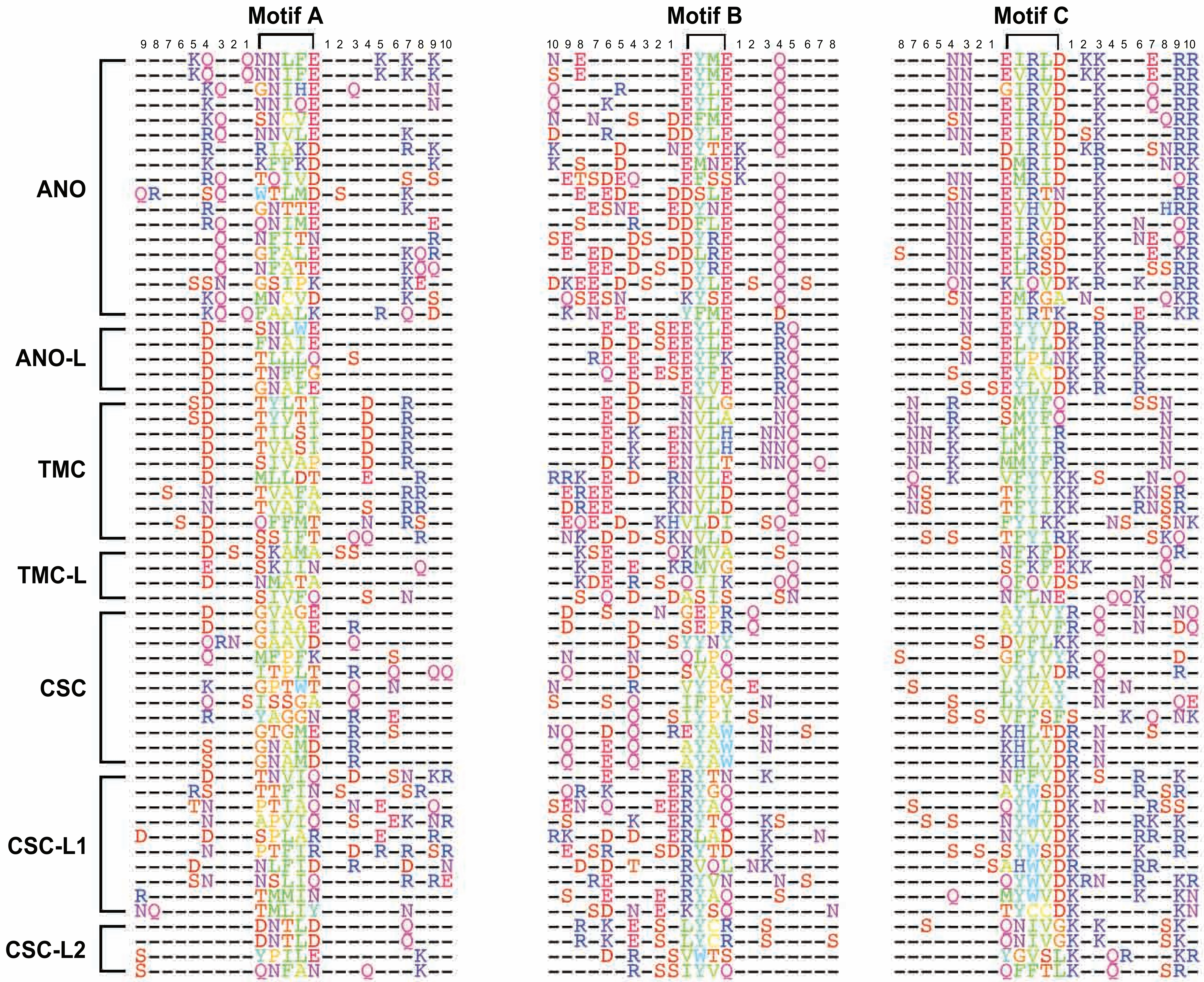
Motif A
Motif B
Motif C
ANO
ANO-L
TMC
TMC-L
CSC
CSC-L1
CSC-L2
